# Identification and expression analysis of heat‐shock proteins in wheat infected with powdery mildew and stripe rust

**DOI:** 10.1002/tpg2.20092

**Published:** 2021-03-14

**Authors:** Huan Guo, Hong Zhang, Guanghao Wang, Changyou Wang, Yajuan Wang, Xinlun Liu, Wanquan Ji

**Affiliations:** ^1^ State Key Laboratory of Crop Stress Biology for Arid Areas, College of Agronomy Northwest A&F University Yangling Shaanxi 712100 P.R. China; ^2^ China‐Australia Joint Research Center for Abiotic and Biotic Stress Management Northwest A&F University Yangling Shaanxi 712100 P.R. China; ^3^ Shaanxi Research Station of Crop Gene Resources and Germplasm Enhancement Ministry of Agriculture Yangling Shaanxi 712100 P.R. China

## Abstract

Heat‐shock proteins (HSPs), which are encoded by conserved gene families in plants, are crucial for development and responses to diverse stresses. However, the wheat (*Triticum aestivum* L.) HSPs have not been systematically classified, especially those involved in protecting plants from disease. Here, we classified 119 DnaJ (Hsp40) proteins (TaDnaJs; encoded by 313 genes) and 41 Hsp70 proteins (TaHsp70s; encoded by 95 genes) into six and four groups, respectively, via a phylogenetic analysis. An examination of protein sequence alignment revealed diversity in the TaDnaJ structural organization, but a highly conserved J‐domain, which was usually characterized by an HPD motif followed by DRD or DED motifs. The expression profiles of HSP‐encoding homologous genes varied in response to *Blumeria graminis* f. sp. *tritici* (*Bgt*) and *Puccinia striiformis* f. sp. *tritici* (*Pst*) stress. A quantitative real‐time polymerase chain reaction (qRT‐PCR) analysis indicated a lack of similarity in the expression of *DnaJ70b*, *Hsp70‐30b*, and *Hsp90‐4b* in *Bgt*‐infected resistant and susceptible wheat. Furthermore, a direct interaction between DnaJ70 and TaHsp70‐30 was not detected in a yeast two‐hybrid (Y2H) assay, but screening cDNA library and Y2H evidence supported that TaHsp70‐30 not only interacts directly with heat‐shock transcription factor (HSF) A9‐like protein but also interacts with TaHsp90‐4 by HSP organizing protein. This study revealed the structure and expression profiles of the HSP‐encoding genes in wheat, which may be useful for future functional elucidation of wheat HSPs responses to fungal infections.

## Core Ideas

- HSPs exhibited structural diversity but DnaJ proteins conserved in J‐domain and DRD–DED motifs.
- The expression profiles of HSP‐encoding homologous genes varied under *Bgt* and *Pst* stress.
- TaHsp70‐30 associates with Hsp90‐4 via a HOP but directly interacts with HSF‐A9L in wheat.

**Abbreviations**
AD: activation domain
BD: binding domain
*Bgt*
: *Blumeria graminis* f. sp. *tritici*
hpi: hours post inoculation
HSF: heat‐shock transcription factor
HSP: heat‐shock protein
NCBI: National Center for Biotechnology Information
*Pst*
: *Puccinia striiform*is f. sp. *tritici*
qRT‐PCR: quantitative real‐time polymerase chain reaction
RNA‐seq: RNA sequencing
SD: synthetic‐defined
Y2H: yeast two‐hybrid.

## 1 INTRODUCTION

In the cytoplasm and nucleus, the heat‐shock response mediates stress‐induced transcriptional changes via the increased production of essential protective factors (Zarouchlioti et al., 2017) called heat‐shock proteins (HSPs; also known as molecular chaperones) (Nollen & Morimoto, 2002). On the basis of their molecular weight, HSPs have been classified into several major families: Hsp90, Hsp70 (also named DnaK in *Escherichia coli*), Hsp40 (also referred to as DnaJ), Hsp60, and the small Hsps. From yeast to humans, Hsp40s and Hsp70s form chaperone partnerships that are key components of cellular chaperone networks involved in facilitating the correct folding of diverse proteins. The DnaJ molecular chaperones, which represent the crucial partners, bind to and transfer substrate proteins to the Hsp70s to regulate their ATPase activity (Ahmad et al., 2011; Qiu et al., 2006). The DnaJ proteins comprise the following four domains: N‐terminal J‐domain, G/F‐domain (Gly/Phe‐rich region), Zn‐binding domain characterized by cysteine repeats, and the C‐terminal dimerization domain (Cuellar et al., 2013; Szabo et al., 1996). Depending on the presence of the G/F‐rich region and the cysteine repeats, a DnaJ–Hsp40 protein is categorized as type I, II, or III (Alderson et al., 2016). The Hsp70–Hsp40–NEF (nucleotide exchange factor) system assists in intracellular protein refolding and helps to maintain proteostasis in healthy and stressed cells (Alderson et al., 2016; Bukau et al., 2006; Papsdorf & Richter, 2014; Weyer et al., 2017). Additionally, Hsp70 functions cooperatively with the highly conserved Hsp90 via cochaperones (Alvira et al., 2014; Benbahouche Nel et al., 2014), resulting in the assembly, maturation, stabilization, and activation of key signaling proteins including protein kinases and transcription factors in eukaryotic cells (Kadota & Shirasu, 2012; Pearl & Prodromou, 2006). These observations suggest there is an important cycle of functions related to the interaction between Hsp70 and the J‐domain proteins Hsp40 and Hsp90 (Murphy, 2013; Ranek et al., 2018). The Hsp90 molecular chaperone also ensures proteins are maintained in their active conformations (Zuehlke et al., 2017).

Plants have no capacity to escape from adverse environments, and their growth and production are severely affected by abiotic and biotic stresses. To ensure they can successfully propagate, plants have developed multiple defense mechanisms that allow them to detect pathogens and induce rapid responses through innate immunity surveillance systems (Nejat & Mantri, 2017; Saijo et al., 2018). Genes encoding HSPs are reportedly differentially expressed in various plant species exposed to abiotic and biotic stresses. During plant‐pathogen interaction, the *AtHsp90.1* gene is required for the full *RESISTANT TO P. SYRINGAE 2* (*RPS2*)‐mediated resistance against *Pseudomonas syringae* pv. *tomato* DC3000 (*avrRpt2*), and its function is closely associated with *RAR1* (required for Mla12 resistance) and *SGT1* (suppressor of the G2 allele of skp1) via chaperone activities (Takahashi et al., 2003). Similarly, suppressing *TaHsp90.2* or *Hsp90.3* expression in wheat (*Triticum aestivum* L.) decreases the hypersensitive resistance to the stripe rust fungus (Wang et al., 2011). Previous studies also confirmed that Hsp70s and Hsp40s are involved in various plant disease resistance, such as NtMPIP1 (Shimizu et al., 2009), GmHSP40.1 (Liu & Whitham, 2013), and OsDjA6 (Zhong et al., 2018). The importance of the proteins encoded by these genes in responses to various environmental stimuli (Landi et al., 2019; Sung et al., 2001; Wen et al., 2017) and their dynamic interplay with the chaperone machinery suggest that targeting Hsp90 and its respective cochaperones may be an effective method for characterizing the mechanisms underlying the resistance to diverse plant diseases.

We previously constructed two protein–protein interaction networks based on a weighted gene coexpression network analysis to clarify the mechanism mediating wheat responses to the pathogens causing stripe rust (*Puccinia striiformis* f. sp. *tritici*; *Pst*) and powdery mildew (*Blumeria graminis* f. sp. *tritici*; *Bgt*) (Hu et al., 2020; Zhang et al., 2019a). Both of these networks predicted that Hsp70 proteins represent a key hub node because they interact with some splicing regulators, transcription factors, and resistance (*R*) genes, including the disease‐resistance‐related *RPP13*, *RPS2* analogues, the pathogenesis‐related protein 1 gene (*PR1*), and a nonhost resistance gene (*NHO1*) (Zhang et al., 2019a). In this study, we identified HSPs and their genes in wheat. Moreover, the expression of these genes following powdery mildew and stripe rust infections was analyzed with RNA sequencing (RNA‐seq) and quantitative real‐time polymerase chain reaction (qRT‐PCR) assays. Furthermore, the interactions among selected HSPs were assessed in a yeast two‐hybrid (Y2H) assay.

**Core Ideas**

- HSPs exhibited structural diversity but DnaJ proteins conserved in J‐domain and DRD–DED motifs.
- The expression profiles of HSP‐encoding homologous genes varied under *Bgt* and *Pst* stress.
- TaHsp70‐30 associates with Hsp90‐4 via a HOP but directly interacts with HSF‐A9L in wheat.

## 2 MATERIALS AND METHODS

### 2.1 Plant materials and pathogen stress treatment

The winter wheat line N9134, which is resistant to all *Bgt* races in China, was backcrossed seven times with a susceptible recurrent parent, Shaanyou225 (SY225) to obtain a resistant line with the SY225 background. This new resistant line was named SY225/7*PmAS846. To eliminate the influence of inhomogeneity, a BC_7_F_1_ resistant plant was self‐crossed to generate the contrasting BC_7_F_2_ homozygous lines, which differ only regarding *PmAS846* on chromosome 5BL and named N9134R and N9134S. Additionally, *Bgt* isolate E09 was maintained on SY225 wheat plants. The contrasting recombinant inbred lines, N9134R/S and SY225 plants, were cultivated in soil in a growth chamber at 18 °C with a 16‐h light vs 8‐h dark photoperiod. Seedlings at the three‐leaf stage were inoculated with *Bgt* conidia from SY225 seedlings that were inoculated 10 d earlier. The inoculated N9134R/S leaves were harvested at 6, 12, 24, 36, 48, 72, and 96 h post inoculation (hpi), after which they were immediately frozen in liquid nitrogen and stored at −80 °C. Subsequent analyses were completed with three biological replicates. For the genome‐wide transcription analysis, 7‐d‐old seedlings were inoculated with *Bgt* E09 or *Pst* race CYR 31 conidia. The inoculated N9134 leaves were harvested at 0, 24, 48, and 72 hpi as previously described (Zhang et al., 2014). The noninoculated sample (0 hpi) was used as the control.

### 2.2 Identification and sequence analyses of wheat heat‐shock proteins

To obtain detailed information regarding wheat HSPs, we downloaded all available sequences for proteins annotated with ‘Hsp’ in the EnsemblPlants database (http://plants.ensembl.org/Triticum_aestivum/Info/Index). After screening the National Center for Biotechnology Information (NCBI) database with the Protein Basic Local Alignment Search Tool (BLASTP) algorithm for sequence matches (80% identity as the threshold), the HSP proteins were sorted and classified. Redundant sequences were manually removed and the remaining sequences were analyzed with the NCBI Conserved Domain database (http://www.ncbi.nlm.nih.gov/Structure/cdd/wrpsb.cgi) and the Pfam database (http://pfam.xfam.org) to detect conserved protein domains and identify candidate *HSP* genes. The motifs of the encoded HSP proteins were detected using the motif‐based sequence analysis program (MEME) (Bailey et al., 2009), whereas a phylogenetic neighbor‐joining tree was constructed for the HSP proteins with the molecular evolutionary genetics analysis (MEGA) program (version 6.0) (Tamura et al., 2013).

### 2.3 Quantitative real‐time PCR analysis

The differentially expressed HSP‐encoding genes that involved in wheat responses to *Bgt* and *Pst* infections were screened using the RNA‐seq data (PRJNA243835) from the fungus‐inoculated N9134 (resistant to *Bgt*‐E09 with *PmAS846* and CYR31 with *YrN9134*). The detailed RNA‐seq protocol was previously described in Zhang et al. (2014). The reads per kilobase of exon model per million of aligned readings (RPKM) values were used to examine the gene expression level distribution for each transcription in sample. In addition, the expression profiles of three specific HSP‐encoding genes in the infected wheat leaves of the contrasting near‐isogenic lines were analyzed by a SYBR Green‐based qRT‐PCR with cDNA prepared from samples collected at 6, 12, 24, 36, 48, 72, and 96 hpi. The uninoculated plant samples at the same time points were set as the controls. Three independent biological replicates were prepared for each time point. The qRT‐PCR was completed with the FastKing RT Kit (with gDNase) (TIANGEN Biotech) and the QuantStudio 7 Flex Real‐Time PCR System (Life Technologies Corporation). Primers specific for the examined HSP‐encoding genes (Supplemental Table S1) and *TaActin* (i.e., internal reference) were designed with the Primer 5 program and were used to analyze gene expression levels. The 20‐μL reaction volume comprised 10 μL 2 × SYBR Green PCR Master Mix (Takara), 0.2 μM each primer, and 2 μL template (6 × diluted cDNA from leaf samples). The PCR program was as follows: 95 °C for 10 s; 40 cycles of 95 °C for 5 s, and 60 °C for 31 s. For each sample, reactions were completed in triplicate, with three nontemplate negative controls. Amplified products were analyzed with melting curves, which were generated at the end of the amplification. A standard 2^−ΔΔCT^ method was used to quantify relative gene expression levels.

### 2.4 Transcriptional activity analysis and yeast two‐hybrid assays

The expression of *HIS3*, *ADE2*, *AUR1‐C*, and *MEL1* reporter genes was examined with yeast Y_2_HGold transformants on SD/−Trp/−His/−Ade/‐Leu media (Clontech Inc.). The fix composition of synthetic‐defined (SD) plates contained 0.67 g yeast nitrogen base, 2 g glucose, 2 g agar, and 0.06 g Do supplement−Trp/−His/−Ade/‐Leu in 100 ml volume. The full‐length open reading frame of *TaHsf‐B1b* as well as the N‐terminal domain and the C‐terminal regulatory domain were amplified by PCR with specific primers containing a homologous recombination arm (Supplemental Table S1). To easily evaluate transcriptional activity, the transformants were suspended in sterile distilled water, then 10‐fold serial dilutions were prepared. Finally, 3‐μl aliquots of each dilution were used to inoculate SD/−Trp/−His/−Ade medium and SD/−Trp/−Leu medium with X‐α‐Gal (Clontech). The inoculated media were incubated for 4 d at 30 °C. The MatchMaker Y2H system (Takara) was used to evaluate the interactions among TaHsp70‐30b, TaHsp90‐4b, and TaDnaJ70b. The *TaHsp70‐30b*, *TaHsp90‐4b*, and *TaDnaJ70b* coding sequences were subcloned into the pGBKT7 (DNA‐binding domain, BD) and pGADT7 (activation domain, AD) vectors. Additionally, the pGADT7‐Sfi I three‐frame primary cDNA library was constructed by Takara (Dalian, China) using leaf samples from N9134 after infected with stripe rust fungi at 24 and 48 h. The AD library was screened for protein interactions by mating pGBKT7:TaHsp70‐30b bait plasmid, while specific AD and BD recombinant plasmid pairs were used to cotransform yeast strain Y_2_HGold cells according to the Yeast Protocols Handbook (Takara).

### 2.5 Statistical analysis

Mean values and standard errors were calculated with Microsoft Excel software. Student's *t* test was completed with the SPSS 16.0 program to assess the significance of any differences between the control and treated samples or between time points. The threshold for significance was set at *P* < .01.

## 3 RESULTS

### 3.1 Identification of wheat heat‐shock protein 40 (DnaJ) family proteins

**FIGURE 1:**
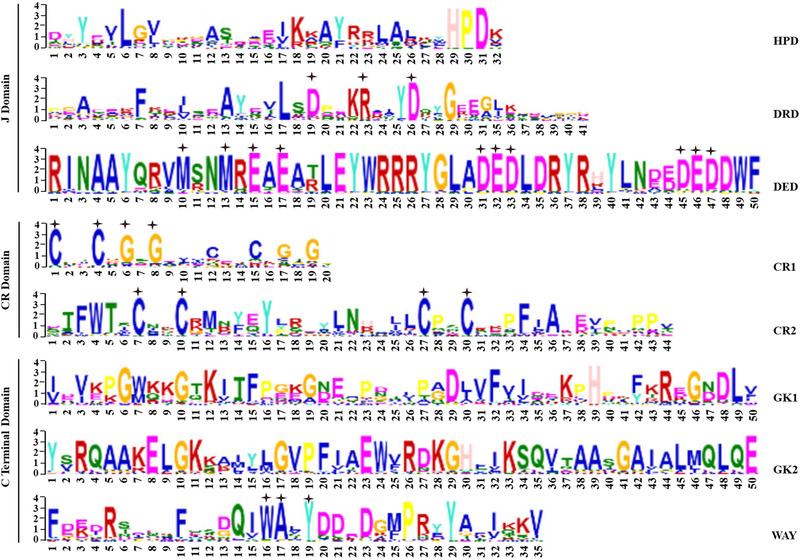
The conserved functional domains and motifs in DnaJ proteins of wheat. The characterization of amino acids were identified by multiple alignment using MEME software. The conserved amino acids of motifs were marked with plus symbol above the letters. The name of motifs were indicated at the right of each sequence. J domain: KKAYRRLALKY**HPD**; DRD domain: AEEKFQEIQEAYEVLS**D**PEK**R**ALY**D**QYG; DED domain: RINAAYQRV**M**SN**M**R**E**A**E**ATLEYWRRR YGLA**DED**LDRYRHYLNDE**DED**DWF; CR domain 1: **C**PT**C**R**G**S**G**EVV**C**DT**C**N**G**T**G**G; CR domain 2: FWTS**C**NK**C**RMNYZYPREYLNRALL**C**PS**C**RK; GK zipper 1: IDVKP**G**WKK**G**T**K**ITFPGK**G**BEAPD; GK zipper 2: **GK**KAIYL**G**VPFIAEWVRD**KG**HFI**K**SQVTAAS**G**; WAY domain: **F**DED**R**SEEK**F**QSDQI **WA**V**Y**DD EDGMPRY**Y**ARIKKV

In order to elucidate the mechanism mediating wheat resistance to *Pst* and *Bgt*, we recently predicted the key genes based on a weighted gene coexpression network analysis and a transcriptome–proteome associated analysis. The predicted genes included TraesCS5B02G374900, which had the most significant connectivity to other genes (value reaching 2,676) (Zhang et al., 2019a) and was annotated as encoding a DnaJ‐like protein. Screening the *PmAs846* physical map, with the ‘Chinese Spring’ wheat genome used as a reference sequence, indicated that TraesCS5B02G374900 is located between the cosegregation markers *BJ261635* with *XFCP620* flanking *PmAS846* (Xue et al., 2012). To further evaluate the genomic distribution and function of HSPs in wheat defenses against pathogens, RNA‐seq data were examined for a genome‐wide identification of HSP‐encoding genes in wheat including genes responsive to fungi. The resulting data confirmed the presence of 119 genes encoding proteins with a characteristic J‐domain in the wheat genome as well as 13 genes for proteins with cysteine repeats without a J‐domain and 12 genes for proteins with a J‐domain but lacking an HPD motif. These three gene groups were designated as *TaDnaJ*, *TaDnaJ‐CR*, and *TaDnaJL*, respectively. These proteins were encoded by 376 wheat genes. Specific details are provided in Supplemental Table S2. Interestingly, the J‐domain was usually accompanied by a domain with the DXXXRXXXD motif or a long‐DED repeat motif (RRRYGLADEDLDRYRXYLNXXDEDDWF) (Figure 1). The DnaJ C‐terminals usually harbored a GK‐rich domain characterized by a glycine and lysine interval zipper or a domain with a WAXY motif.

**FIGURE 2:**
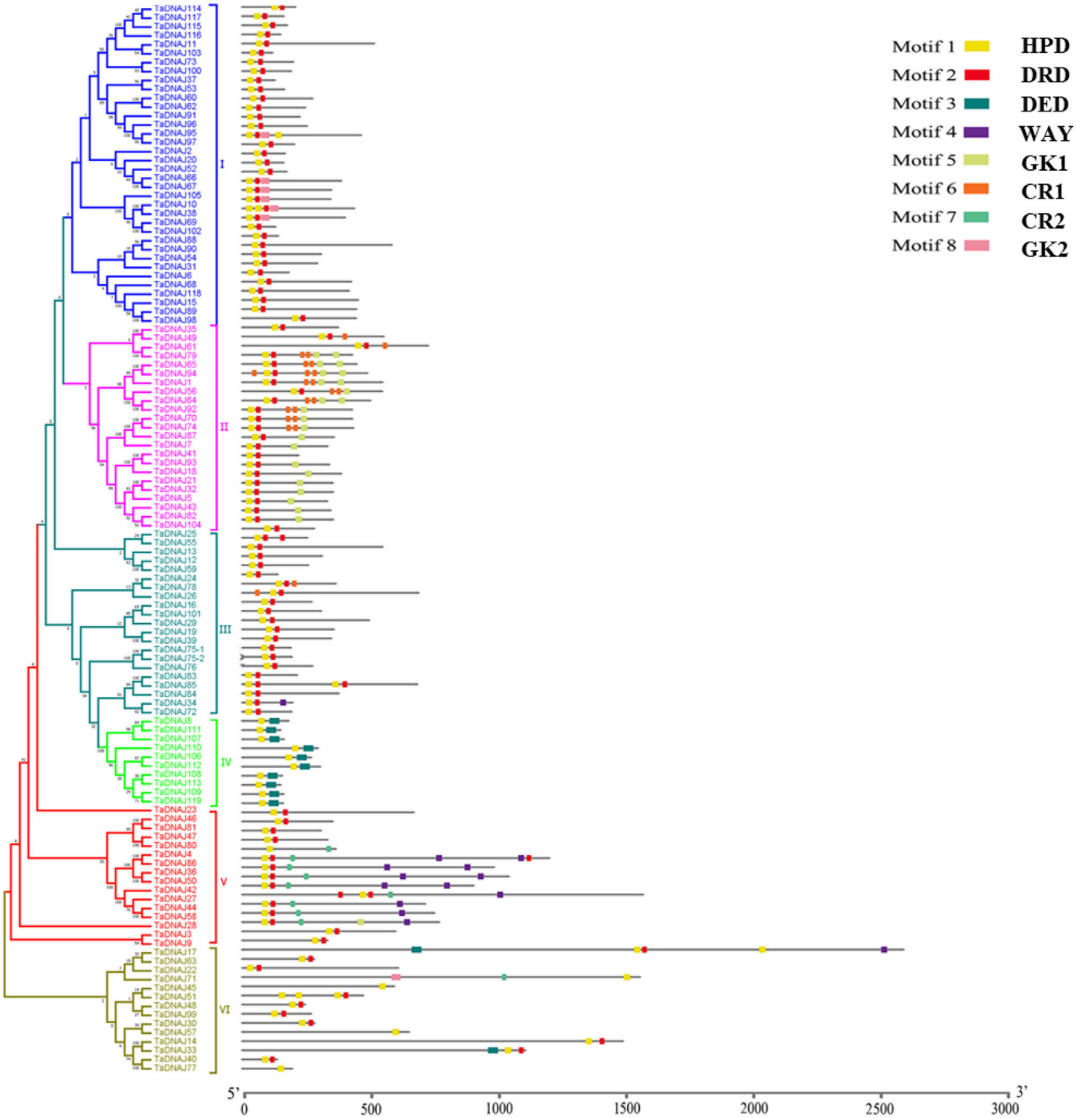
Phylogenetic tree, conserved motifs, and sequence structure of 119 TaDnaJ proteins. The conserved motifs were listed as differential colored box. The sequence structure of TaDnaJ proteins were represented with the mixed thin line and color boxes, while the scale was given in the bottom of panel

According to the present classification of DnaJ, the N‐terminal J‐domain of class I DnaJ proteins was followed by a G/F‐rich region, four repeats of the CXXCXGXG‐type zinc‐finger, and a C‐terminal extension (Kampinga & Craig, 2010). However, sequence alignment results indicated that only 17 wheat DnaJ proteins (not including 13 CR domain‐containing DnaJ proteins) had a cysteine repeat domain. Of these proteins, nine comprised four repeats of CXXCXGXG and the other eight proteins consisted of two repeats of the CXXC motif. Similarly, only 17 proteins contained a G/F‐domain, which was replaced by AP‐, SP‐, AS‐, GS‐, or GR‐rich domains in the other wheat DnaJ proteins. Additionally, we detected 119 wheat DnaJ proteins that were encoded by 313 genes (Supplemental Table S2). We observed that Class II proteins present variable domains, whereas most of the Class III proteins consist of domains with characteristic DRD or DED motifs. This means that large structural and potential functional differences exist within and between traditional Classes II and III of DnaJ. Considering the fact that the presence (type II) or absence (type III) of the G/F‐domain has led to some ambiguity (Kampinga & Craig, 2010), the 119 wheat DnaJ proteins were further classified in six subfamilies (Figure 2) on the basis of the characterized domains. Type I DnaJ proteins were characterized by a DRD motif, whereas type IV and type V DnaJ proteins harbored the DED and WAY motifs, respectively. The type II DnaJ proteins had a classical zinc‐finger domain. The J‐domain was accompanied by an uncharacterized N‐terminal on the type‐III DnaJ proteins.

### 3.2 Identification of the wheat 70‐ and 90‐kD heat‐shock proteins

**FIGURE 3:**
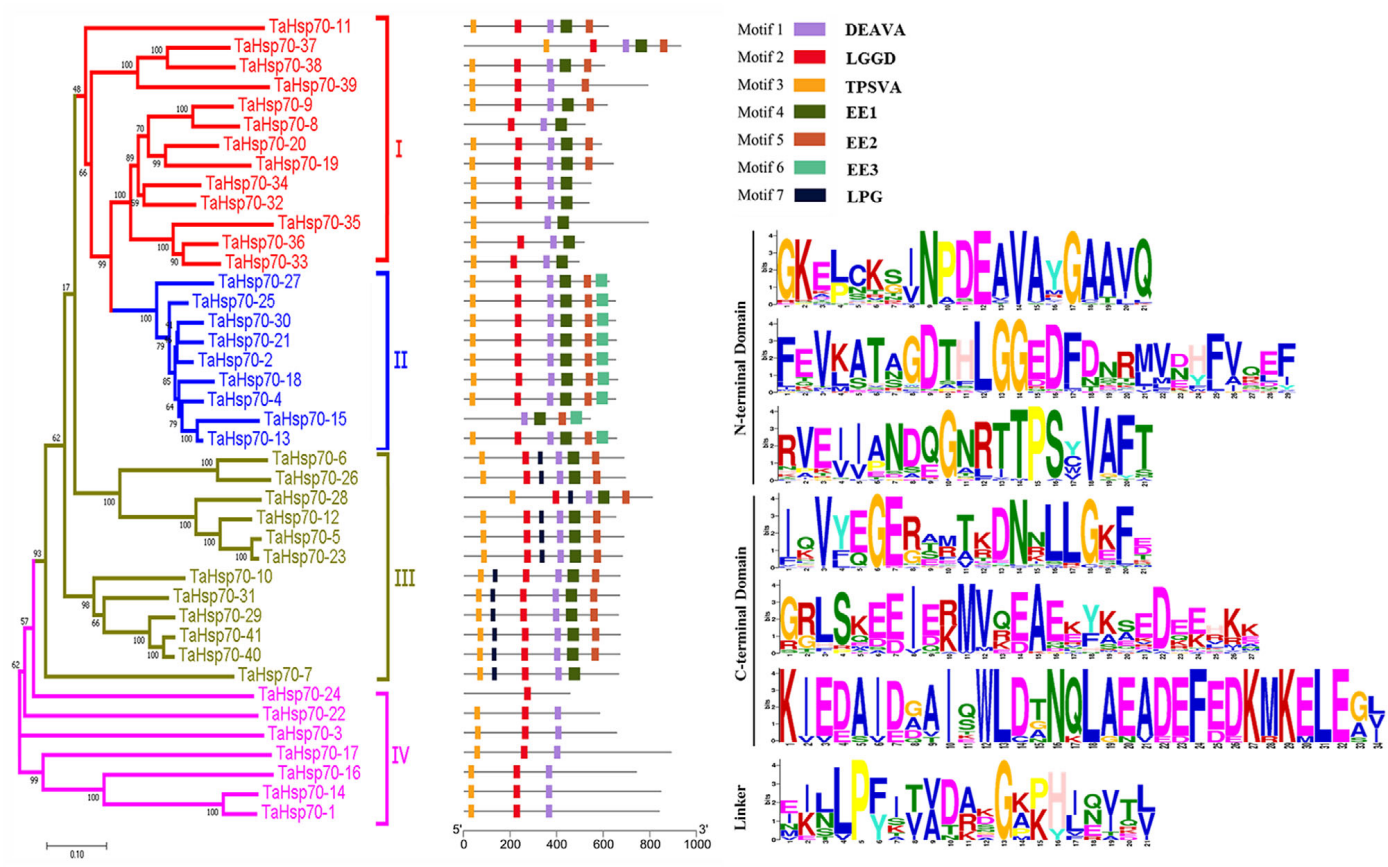
Phylogenetic tree, conserved motifs, and sequence structure of Hsp70 proteins in wheat. The conserved motifs were listed as differential colored box, and their names and sequences were orderly shown in the right. The gene structure of TaHsp70 proteins were represented with the mixed thin line and color boxes, while the scale was given in the left bottom of panel. The characterization of amino acids were identified by multiple alignment using MEME software. Motif 1: GKELCKSI**N**P**DEAVA**YGAAVQ; motif 2: FEVKATAG**D**TH**LGG**E**D**FDNRMVDHFV; motif 3: RVEIIANDQGNRT**TPS**Y**VAF**TDTER; motif 4: IQVY**EGE**RAMTKDNNLLGEFE; motif 5: GRLSKE**EIE**KMVQ**EAE**KYKS**EDE**EHKK; motif 6: KIEDAIDGAIQWLDTNQLAEAD**EFE**DKMK**ELE**GVCNP; motif 7 (seem to be linker): EIL**LP**FITVDAK**G**KPHIQVTL

We detected 41 Hsp70 proteins (encoded by 95 genes) as well as two proteins containing partial Hsp70 domains, two Hsp70‐like proteins, and six Hsp90 proteins (encoded by 18 genes) in the wheat reference genome. Specific details are listed in Supplemental Tables S3 and S4. In wheat, the Hsp70 proteins are more conserved than the DnaJ proteins. The six domains detected in 41 unabridged Hsp70 proteins are presented in Figure 3. Of these domains, the NXDEAVA, DXXLGGXD, and TPSXVAF motifs were detected in nearly all members. The repetition of the C‐terminal EXE motif (wherein X represents glycine, isoleucine, alanine, aspartic acid, leucine, or phenylalanine) was used to classify the Hsp70 proteins in four subfamilies (Figure 3). The type IV Hsp70 proteins lacked the EXE motif, whereas the type II Hsp70 proteins had more EXE repeats than the type I and type III proteins. Interestingly, the type II Hsp70 proteins contained an EEVD pattern at the C‐terminal, unlike the type III proteins, which comprised a C‐terminal HDEL pattern. Another difference between the type I and type III proteins was the presence of the nontypical linker motif 7 in the type III proteins (Figure 3). We detected far fewer Hsp90 proteins than Hsp70 and DnaJ proteins. However, the wheat Hsp90 proteins were observed to contain very similar ED‐enriched domains, usually accompanied by a leucine zipper.

### 3.3 Expression of heat‐shock protein‐coding genes in wheat–*Bgt* and wheat–*Pst* interactions

**FIGURE 4:**
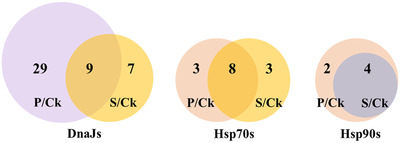
Venn diagram for the overlap differentially expressed Hsp‐encoding genes identified with *Bgt* and *Pst* treatments. The number of specific and overlapped genes were given

**FIGURE 5:**
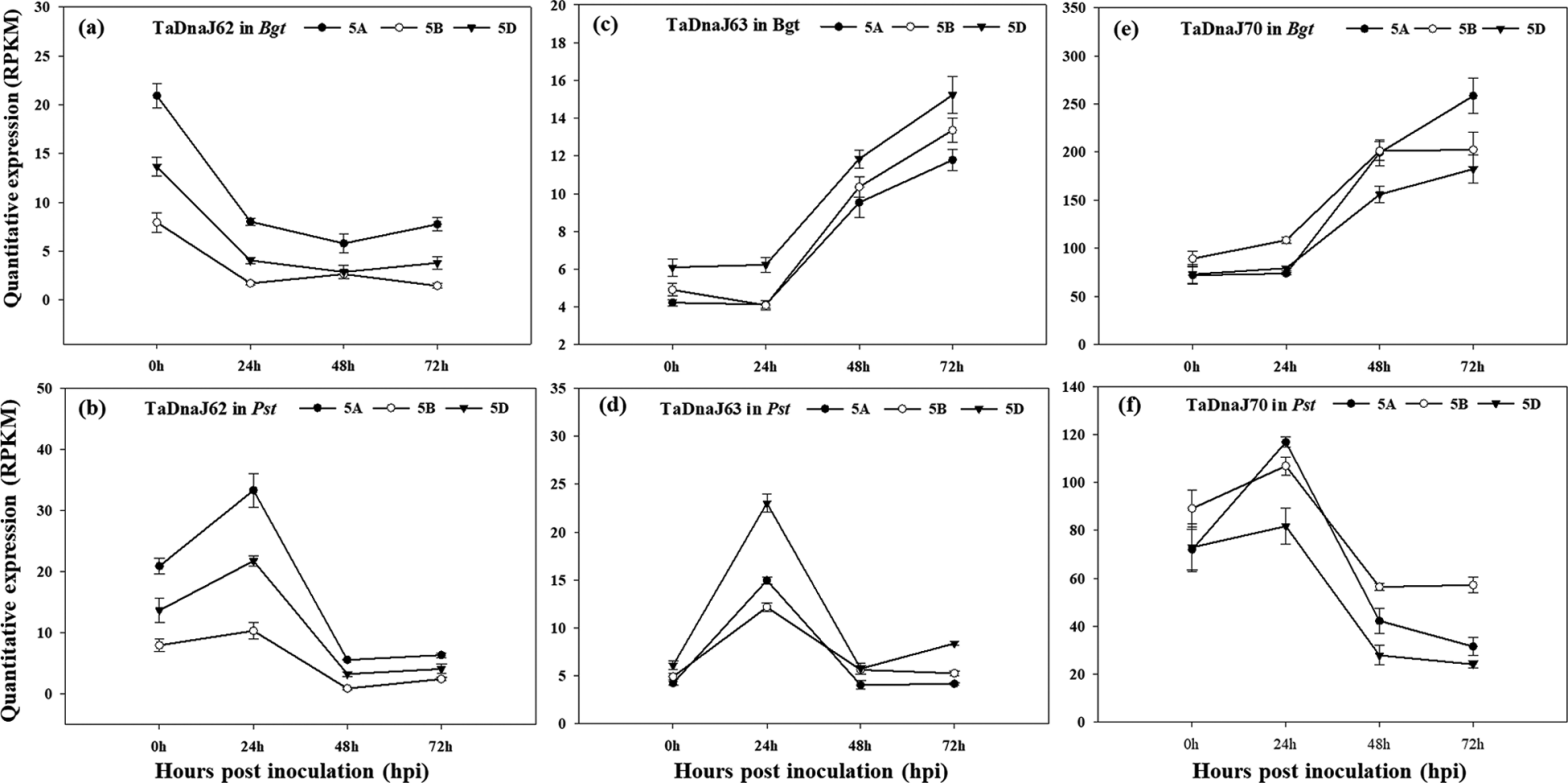
Expression patterns of differentially expressed DnaJ‐encoding genes in N9134 infected by stripe rust and powdery mildew pathogen. Gene expression levels were assessed by transcript accumulation analysis. The mean expression value was calculated from three independent replicates. Line charts mean the gene expression of homologue HSP‐encoding genes in wheat infected by stripe rust and powdery mildew. The number 0, 24, 48, and 72 indicated the timepoints after infection. (a), (c), and (e) represent *Bgt* E09 inoculation condition; (b), (d), and (f) represent *Pst* CYR 31 inoculation. The name of DnaJ‐encoding genes and corresponding chromosome were listed in the top of each panel

To identify the HSP‐encoding genes involved in wheat responses to *Bgt* and *Pst* infections, we screened the RNA‐seq data (PRJNA243835). Based on the RNA‐seq data of N9134 in *Bgt* and *Pst* treatments, the expression heatmap of *TaDnaJ*, *TaHsp70*, and *TaHsp90* on chromosomes A, B, and D were made by TBtools after simply eliminating the nonexpressed genes (Supplemental Figure S1a–c and S2a–c). Interestingly, the expression trends of these A, B, D homologous genes were quite similar in different periods of infections, but the expression patterns of these genes were different under the infections of *Bgt* and *Pst*. Under the stress of *Bgt*, two thirds of *TaDnaJs* expression had no change at 24 hpi but increased after 48 and 72 hpi, while these genes expressed monotonously in *Pst* stress. A part of other *TaDnaJ* genes expression increased 48 and 72 hpi after *Pst* stress. Most expression of *Hsp70* and *Hsp90* were highest in 24 hpi after infection with *Bgt* and then decreased slowly. It was surprising that almost all gene expressions were inhibited at 24 hpi, recovered slowly at 48 hpi, then increased significantly at 72 hpi. These indicated that the response patterns of stress genes are different between the infection of *Bgt* and *Pst*. In 10‐d‐old N9134 seedlings inoculated with *Bgt*, 45 *DnaJ* family genes encoding 20 HSPs were identified as differentially expressed genes (relative to the control expression level) (Figure 4; Supplemental Table S2). Moreover, 14 *Hsp70* genes (encoding seven Hsp70 proteins) and six *Hsp90* genes (encoding three Hsp90 proteins) were identified in the fungus‐inoculated N9134 seedling leaves (Supplemental Tables S3 and S4). Notably, the number of DnaJs was three times more than that of Hsp70s, while it was near to seven times than the number of Hsp90s. Conversely, the ratio of the overlapped genes was steeply increased, which reached 20, 57.1, and 66.7% for DnaJs, Hsp70s, and Hsp90s, respectively. Considering wheat comprises a polyploid genome, we further analyzed the differentially expressed genes encoding the same HSP on partially homologous chromosomes. The results indicated that the differentially expressed *Hsp* genes from the partially homologous chromosomes had nearly coincident expression patterns, although the expression levels differed (Figure 5; Supplemental Figure S3). For example, the expression of *TaDnaJ7‐1A*, *TaDnaJ7‐1B*, and *TaDnaJ7‐1D* increased at 24 hpi in *Bgt*‐infected plants, after which the expression sharply decreased at 48 and 72 hpi (Supplemental Figure S3). The transcription levels of *TaDnaJ7‐1A* and *TaDnaJ7‐1D* were 2.5‐ and 2.2‐fold higher than that of *TaDnaJ7‐1B*, respectively. Additionally, *TaDnaJ86‐6A*, *TaDnaJ86‐6B*, and *TaDnaJ86‐6D* expression levels were not upregulated until 48 hpi, after which they continued to increase, peaking at 72 hpi. Subsequently, we analyzed the level of protein expression from previous quantitative proteomic data (PeptideAtlas: PASS00682 and PASS00999) (Fu et al., 2016; Zhang et al., 2019a), and found that TaDnaJ7, TaDnaJ70, TaHsp70‐4, TaHsp70‐25, TaHsp70‐30, and TaHsp90‐4 proteins were also differentially accumulated with >1.2‐fold change in abundance and *t* test *p* value <.05 (FDR < 1%) in fungi‐infected leaves comparing with uninfected control.

**FIGURE 6:**
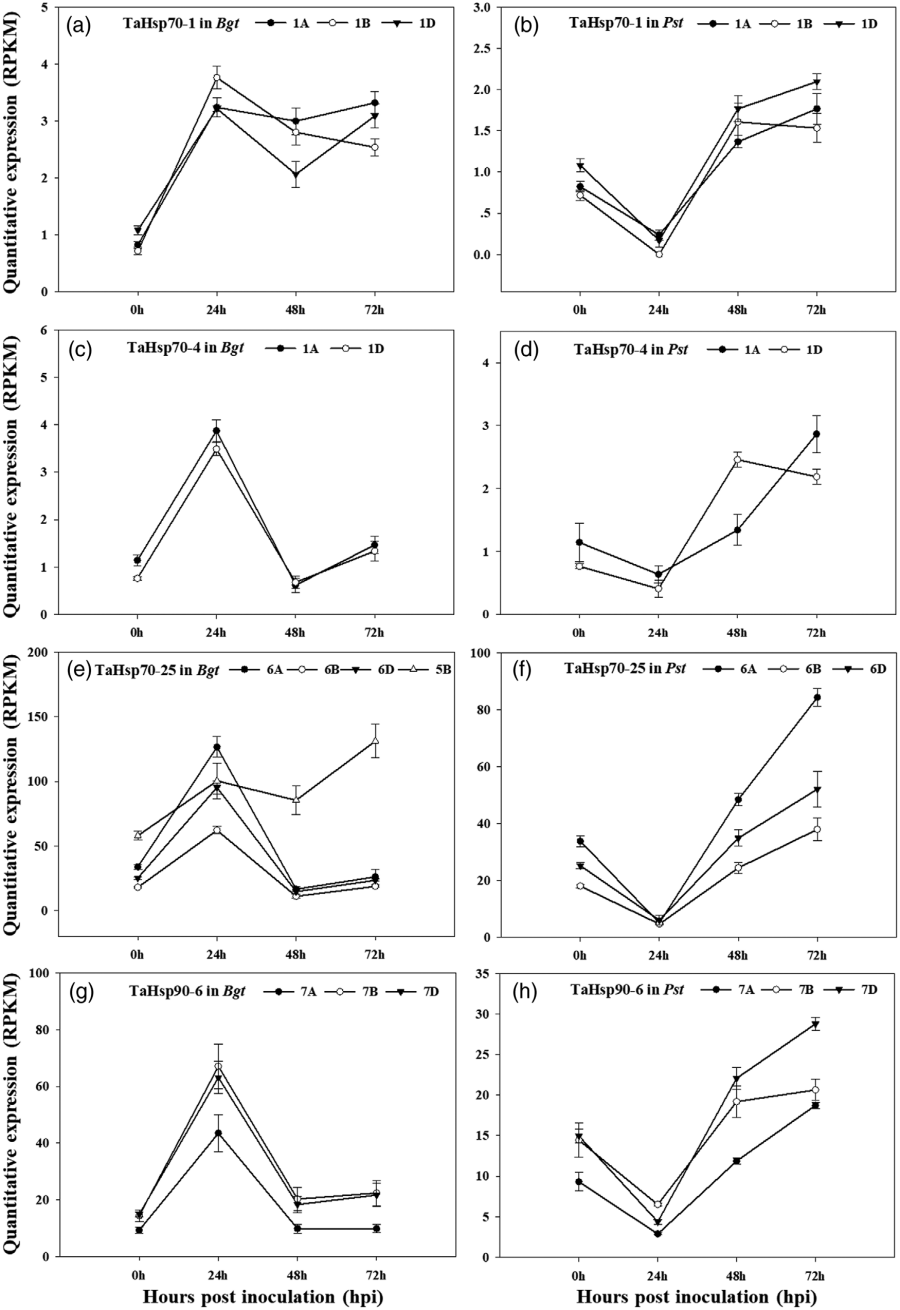
Expression patterns of differentially expressed Hsp70‐ and Hsp90‐encoding genes in N9134 infected by stripe rust and powdery mildew pathogen. Gene expression levels were assessed by transcript accumulation analysis. The mean expression value was calculated from three independent replicates. Line charts mean the gene expression of homologue HSP‐encoding genes in wheat infected by stripe rust and powdery mildew. The number 0, 24, 48, and 72 indicated the timepoints after infection. (a), (c), (e), and (g) represent *Bgt* E09 inoculation condition; (b), (d), (f), and (h) represent *Pst* CYR 31 inoculation. The name of HSP‐encoding genes and corresponding chromosome were listed in the top of each panel

The overlapping differentially expressed *Hsp* genes exhibited the complete opposite expression patterns in response to the *Bgt* and *Pst* infections (Figure 5). Specifically, the transcription of *TaDnaJ62‐5A*, *TaDnaJ62‐5B*, and *TaDnaJ62‐5D* were repressed at 24 hpi in the *Bgt*‐infected plants but induced at the same time point in the *Pst*‐infected plants. The *TaDnaJ70* expression level was upregulated at 48 and 72 hpi in the *Bgt*‐infected plants but downregulated in the *Pst*‐infected plants. The *TaHsp70‐1*, *TaHsp70‐4*, *TaHsp70‐25*, and *TaHsp90‐6* expression levels were induced at 24 hpi in *Bgt*‐infected plants, whereas they were repressed at 24 hpi in the *Pst*‐infected plants (Figure 6; Supplemental Figure S4). These observations supported our previous view that distinct genes and regulatory networks are activated in wheat to counter the adverse effects of *Bgt* and *Pst* infections (Zhang et al., 2014, 2019b).

In addition to the wheat HSPs, we identified three *Bgt*‐*DnaJ* homologues (accession numbers KE373410.1, KE374995.1, and KE375122.1), one *Bgt*‐*Hsp60* (CAUH01004772.1), four *Bgt*‐*Hsp70* (JQ917466.1, EPQ67599.1, EPQ66911.1, and KE375027.1), and two *Bgt*‐*Hsp90* (KE374996.1 and EPQ62547.1) genes (Table 1). These fungal HSP‐encoding genes were detected after plants were infected (Supplemental Figure S5) and had varying expression patterns. The Hsp70‐encoding gene JQ917466.1 and Hsp90‐encoding gene KE374996.1 were most highly expressed at 48 hpi, whereas the expression of the *DnaJ* homologue KE374995.1 stably increased after plants were infected. These results implied that the HSPs may contribute to the fungal infections of susceptible host plants.

**TABLE 1:** The detected Bgt‐HSP encoding genes in N9134 after powdery mildew infection

Encoding protein	Accession no.	Accession no.	Accession no.	Accession no.
Bgt‐DnaJ	KE373410.1	KE374995.1	KE375122.1	–
Bgt‐Hsp60	CAUH01004772.1	–	–	–
Bgt‐Hsp70	JQ917466.1	EPQ67599.1	EPQ66911.1	KE375027.1
Bgt‐Hsp90	KE374996.1	EPQ62547.1	–	–

### 3.4 Coexpression of TaHsp70‐30b, TaHsp90‐4b, and TaDnaJ70b in wheat–*Bgt* interactions

**FIGURE 7:**
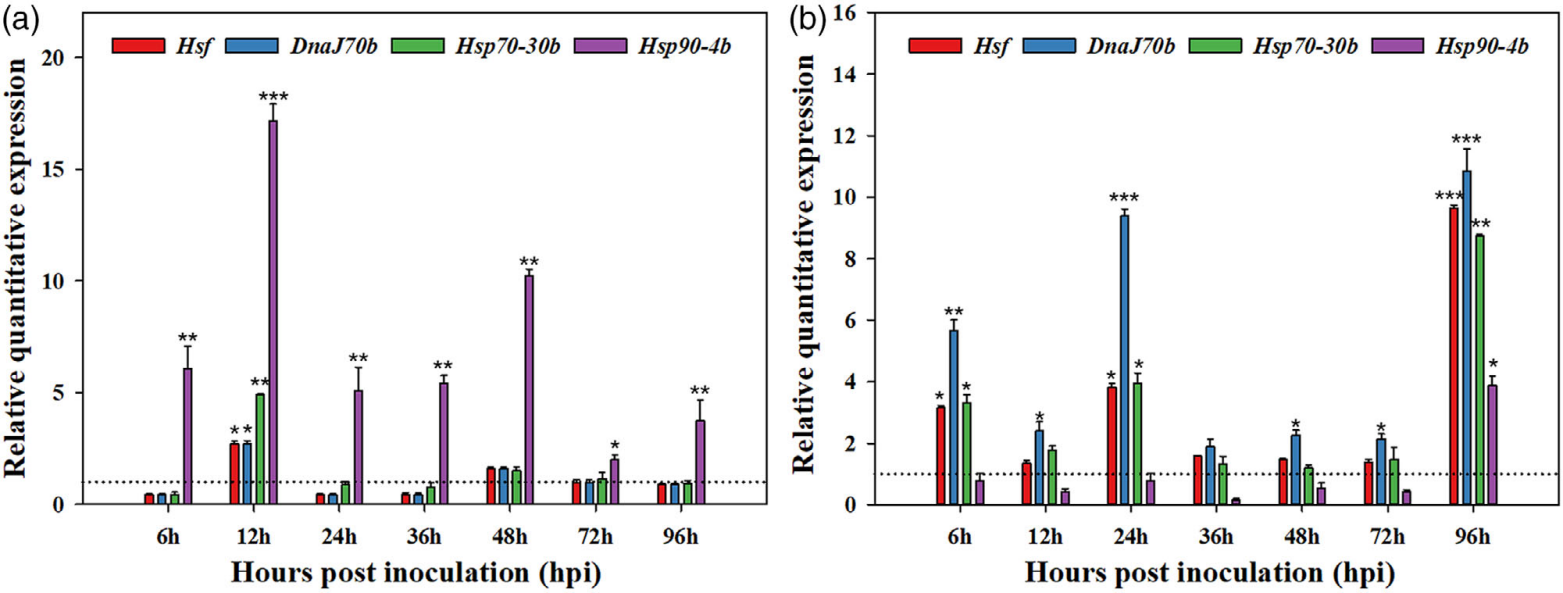
Coexpression patterns of selected heat‐shock transcription factor and HSP‐encoding genes in N9134R and N9134S induction by powdery mildew pathogen. Gene expression levels were assessed by qRT‐PCR at 6, 12, 24, 36, 48, 72, and 96 h post inoculation. Data were normalized to the *actin* expression level. The mean expression value was calculated from three independent replicates, and the standard deviation was given at each time points. (a) represents gene expression in N9134R with resistance to powdery mildew E09; (b) represents gene expression in susceptible line N9134S. The dotted line indicated the controls at the same time points. The names of corresponding gene were listed in the top of each panel

To assess the expression and function of HSP‐encoding genes, *TaDnaJ70b*, *TaHsp70‐30b*, *TaHsp90‐4b*, and a wheat heat‐shock transcription factor (HSF) gene, *TaHsf‐B1b*, all of which are located on chromosome 5B, were analyzed regarding their expression level in the *Bgt*‐infected leaves of resistant line N9134R (SY225/7*PmAS846, a Shaanyou225 backcross line carrying *PmAS846* from N9134) and the contrasting susceptible N9134S near‐isogenic lines. For the sake of preciseness, the cytological observations were carried out at 24, 48, and 72 h after infection of N9134R/S by *Bgt* isolate E09 (Supplemental Figure S6). Because of the repetition in the wheat genome sequence, we used four pairs of specific primers targeting the 5′ terminal sequences to target the genes of interest on chromosome 5B. The expression profiles of *TaHsf‐B1b*, *TaDnaJ70b*, *TaHsp70‐30b*, and *TaHsp90‐4b* in the inoculated N9134R and N9134S plants are presented in Figure 7. Following the inoculation with *Bgt*, the expression levels of all tested genes were significantly dysregulated at 6 hpi, whereas they were oppositely down‐ and upregulated in the N9134R and N9134S plants, respectively. There was a subsequent steep increase in the expression level at 12 hpi in the N9134R plants. The *TaHsf‐B1b*, *TaDnaJ70b*, and *TaHsp70‐30* expression levels at 6 hpi were higher than that in the mock‐inoculated control N9134S plants. Afterward, the high expression levels of *TaDnaJ70b*, *TaHsp70‐30b*, and *TaHsp90‐4b* fluctuated irregularly in N9134S plants, with *TaDnaJ70b* expression peaking at 24 and 96 hpi. Conversely, in N9134R plants, *TaHsf‐B1b*, *TaDnaJ70b*, and *TaHsp70‐30b* were relatively stably expressed at low levels, with only slight and consistent fluctuations from 12 to 96 hpi. The highest *TaHsp90‐4b* expression levels were observed at 96 hpi in N9134S plants, although they were not significantly higher at other time points than the corresponding expression levels in control (Figure 7) with resistance background. Thus, there were no coexpression relationships between *TaHsp90‐4b* with *TaDnaJ70b* and *TaHsp70‐30b*, while the gene expression pattern of *TaHsf‐B1b* was very similar to that of *TaDnaJ70b* and *TaHsp70‐30b*. Additionally, the expression levels of the DnaJ and Hsp70‐encoding genes were generally higher in the susceptible plants than in the resistant plants, but the expression of *Hsp90‐4b* is just the reverse.

### 3.5 In vitro interactions among TaHsp70‐30b, TaHsp90‐4b, and TaDnaJ70b proteins

**FIGURE 8:**
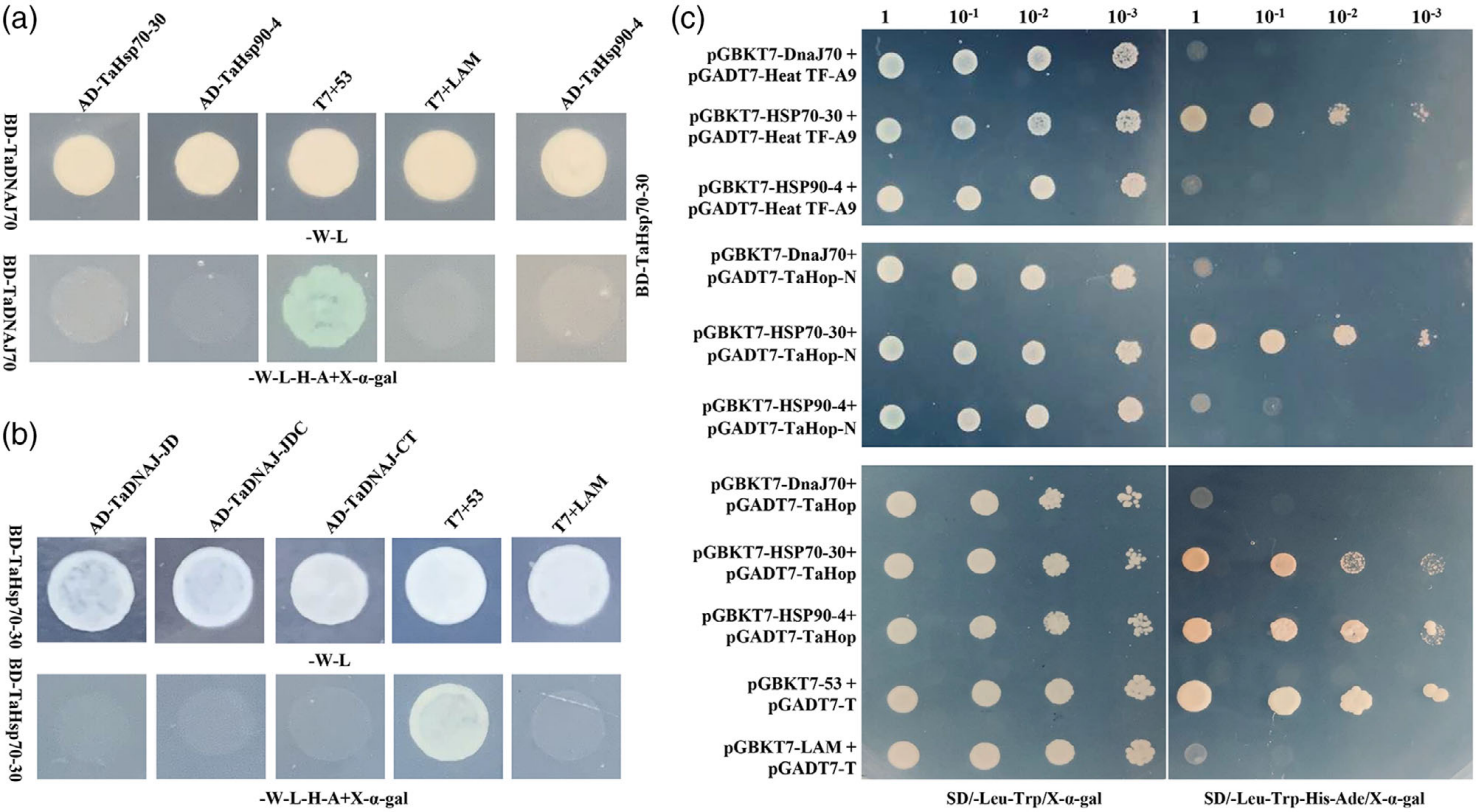
The interaction between TaDnaJ70, TaHsp70‐30, TaHsp90‐4 proteins and their potential interactors in a yeast two‐hybrid system. (a) TaDnaJ70 or (b) TaHsp70‐30 was cloned into pGBKT7 vector and the corresponding proteins were cloned into the pGADT7 vector. Cells of yeast strain Y2H harboring the indicated plasmid combinations were grown on either the nonselective (SD‐L‐W) or selective (SD‐L‐W‐H‐A) medium containing 20 μg ml^−1^ X‐a‐gal. The interaction between SV40 large T‐antigen (T) and murine p53 (53), T‐AD+53‐BD, was used as the positive control, while the interaction between T‐antigen and human lamin C (Lam), T‐AD+Lam‐BD, was used as the negative control. (a) The test of interaction between full length proteins. (b) The test of interaction between full lengths TaHsp70‐30 with the truncated DnaJ70 proteins. JD means the J and DRD domains; JDC represents the J, DRD and zinc finger domains, while CT means the C‐termianl of TaDnaJ70. (c) TaHsp70‐30 and TaHsp90‐4 interact with TaHop and Hsf A9 directly

The *TaHsp90.2* and *TaHsp90.3* genes, located on the second and seventh partially homologous chromosomes, respectively, are involved in wheat responses to the stripe rust pathogen (Wang et al., 2011). In *Arabidopsis thaliana* (L.) Heynh., The J‐domain of DnaJ proteins reportedly interacts with Hsp70. Previously, TaDnaJ70, TaHsp70‐30, and TaHsp90‐4 were detected as specific induced genes in fungal stress (Zhang et al., 2019a). To understand the roles of HSPs in wheat resistance, we tested the interaction between them using the Y2H system. After the expression of the proteins of interest in the Y2H system was confirmed on SD medium lacking Trp and Leu (double dropout, DDO), the yeast strains with BD‐ and AD‐TaHsp were grown on SD/−Trp/−Leu/−His/−Ade medium (quadruple dropout, QDO). The TaDnaJ70 protein, either in full or with a truncated N‐/C‐terminal, failed to interact with TaHsp70‐30 in the Y2H assay (Figure 8a and 8b). Similarly, TaHsp90‐4 did not interact directly with TaDnaJ70 or TaHsp70‐30 (Figure 8a).

To capture Hsp70 interactors, we screened a cDNA library of *Bgt*‐infected N9134 using bait and identified two proteins. After the plasmid was extracted, amplified, sequenced, and comparative analyzed, it was found that the two sequences encode HSF A9‐like protein (TraesCS4B02G239700) and 344 amino acids in the N‐terminal of HSP organizing protein (TraesCS6B02G285800) separately. Furthermore, the interactions were evidenced by Y2H between TaHsp70‐30 with HSP organizing protein, as well as between TaHsp70‐30 and HSF A9‐like protein, in the stringent quadruple dropout medium even at the 1:1000 dilution, while no interaction was found between TaHsp90‐4 and N‐terminal of HSP organizing protein (Figure 8c). Then we amplified the full length of HSP organizing protein from cDNA to study its new interaction by constructing prey vector. What is amazing is that we found that the full‐length HSP organizing protein could interact with both TaHsp70‐30 and TaHsp90‐4. This indicates that TaHsp70‐30 and TaHsp90‐4 participate in protein folding, conformational regulation, and stabilization through binding N‐ and C‐terminal of HSP organizing protein, respectively. Notably, no DnaJ was detected as an interactor of TaHsp70‐30. This hinted that the interaction between DnaJ and Hsp70 may occur selectively or indirectly in wheat (e.g. via an adapter).

## 4 DISCUSSION

### 4.1 DnaJ protein groups, structure, and classification

Historically, DnaJ proteins have been divided into three classes (I, II, and III) based on the G/F and zinc‐finger motifs and domains present in *Escherichia coli* DnaJ proteins (Kampinga & Craig, 2010). Obviously, the large structural and potential functional differences exist within and between Classes II and III. In this study, we detected 144 DnaJ family proteins, but only nine out of 119 DnaJ have the classical CXXCXGXG domain. This domain was replaced by a CXXC repeat in 12 DnaJ proteins, whereas the classical domain exists in 10 out of 13 DnaJ‐CR proteins that lack the J‐domain. Additionally, some of the proteins have similar N‐terminal J‐domain sequences but are missing the aspartic acid in the HPD motif. For clarity, we refer to these domains as ‘J‐like.’ To clarify the biochemical functions or mechanisms associated with DnaJ proteins, we further classified these proteins in six groups with characteristic DRD, DED, CR zinc‐finger, or WAY motifs. The current study represents the first systematic analysis of wheat genes encoding Hsp40 and Hsp70 proteins and generated useful information regarding the expression of HSP‐encoding genes of common wheat. The presented data may form the basis for future investigations into the genome‐wide *DnaJ* and *Hsp70* expression dynamics during plant–pathogen interactions. Furthermore, our findings may be relevant for further elucidating the role of HSPs in wheat defense responses as well as the detailed effects of the diverse HSP domains.

### 4.2 Heat‐stress proteins play a critical role in wheat responding to pathogen

Heat‐shock proteins [e.g., Hsp40 (DnaJ), Hsp60, Hsp70, Hsp90, and Hsp101], which form one of the most ubiquitous classes of chaperones, have been implicated in diverse biological processes. The HSPs also directly stimulate cells of the innate immune system, suggesting they are activators of the innate immune system in animals (Cui et al., 2011; Wallin et al., 2002). For example, a nonlethal heat shock induces Hsp70 synthesis and promotes the tolerance of shrimp (*Penaeus vannamei*) to stresses resulting from heat, ammonia, and metals (Sung et al., 2018) and also prevents heart failure or ageing in humans (Ranek et al., 2018). So far, several plant species’ Hsp70s have been surveyed, such as barley (*Hordeum vulgare* L.) (Landi et al., 2019), *Brachypodium distachyon* (L.) Beauv. (Wen et al., 2017), *A. thaliana* (Leng et al., 2017), and California poplar (*Populus trichocarpa* Torr. & A. Gray) (Yer et al., 2018). Plant Hsp70s are localized to the cytosol, endoplasmic reticulum, mitochondria, chloroplasts, and peroxisomes (Sung et al., 2001). Although there have been limited functional studies on plant Hsp70s, these proteins are believed to have multiple roles in plants and function like other eukaryotic Hsp70s. As for Hsp90s, the function implicated in disease resistance had been very well evidenced because interacting with RAR1, SGT1 (Kadota et al., 2010; Takahashi et al., 2003), tomato I‐2 (de la Fuente van Bentem et al., 2005), NbSIPK (Kanzaki et al., 2003), and others. Unfortunately, the available information related to the functions of HSPs in common wheat and other Triticeae species is still very limited. In our study, most of HSP expression level changed in N9134 infected by *Bgt* and *Pst* indicating that HSPs family actively participated in the stress response to fungi. But notably, the expression profiles of HSPs is different under the stress of these two pathogens means that the function of the same HSP protein in different pathogen stress was different. In wheat, *TaHSC70* encodes a TaHsp70‐26 protein and is responsive to stripe rust infections via a JA‐dependent signal transduction pathway (Duan et al., 2011). The cytosolic Hsp90s contribute to the resistance of wheat to the stripe rust fungus (Wang et al., 2011). We previously identified Hsp70 as a key hub node protein and determined that it likely plays a critical role in wheat defenses against fungal infections (Zhang et al., 2019a). In the current study, we identified 45 differentially expressed *DnaJ* genes as well as 14 *Hsp70* genes and six *Hsp90* genes in the fungus‐inoculated N9134 seedling leaves. One interesting phenomenon is that the number of Hsp90s is the smallest among the differentially expressed genes, and the proportion of overlapping genes is the highest. These results imply that HSPs influence plant resistance to fungi in ways that remain to be determined. Although it is unclear why the expression levels of HSP‐encoding genes are considerably and differentially affected by powdery mildew and stripe rust infections in resistant and susceptible wheat leaves, the data presented herein verify that HSPs have diverse functions in plants. In addition, focused on those genes that the expression have significant changes in pathogen infection but show opposite expression patterns under *Bgt* and *Pst* stress, we could better understand their specific functions of HSPs in plant.

### 4.3 Heat‐stress proteins may indirectly interact with each other

The Hsp90 chaperone pathway involves a series of steps including the formation of multiple‐chaperone complexes with the assistance of receptors and cofactors. After Hsp40 binds to a receptor, its J‐domain interacts with the C‐terminus of Hsp70 (Weyer et al., 2017). The N‐terminal of Hsp70 simultaneously binds to ATP. An intermediate complex, comprised of Hsp70 and Hsp90, is then formed with the assistance of cofactors that help ATP bind to Hsp90 (Cintron & Toft, 2006). In this process, Hsp70 and Hsp90 are associated through the Hop adapter protein (Alvira et al., 2014), which also has been substantiated here. Additionally, because the chaperone partnership between Hsp40s and Hsp70s has been well established from yeast to humans (Fiaux et al., 2010), it was assumed, without experimental verification, that these HSPs also interact in plants. In fact, there are inter‐ and intraspecies variations in the J‐domain, hinting at the specificity of Hsp40–Hsp70 interactions (Hennessy et al., 2005). In wheat, there are 144 DnaJ family proteins (119 DnaJ, 12 DnaJ‐like, and 13 DnaJ‐CR proteins) and 41 Hsp70s, with considerable diversity in both protein families. Thus, it is problematic if these proteins are able to freely interact with each other in wheat plants. In this study, our data indicated that TaDnaJ70 does not directly interact with either TaHsp70‐30 or TaHsp90‐4 or that our technique is not sensitive enough to detect the binding. There are two possible relationships between the DnaJ and Hsp70 proteins. Specifically, DnaJ may directly interact with specific Hsp70s but not arbitrarily in wheat, or the interaction may be indirect (e.g., via an adapter), similar to the interaction between Hsp70 and Hsp90. Because the presence of the HPD motif in TaDnaJ70 has been confirmed, these results imply that other residues and regions outside the HPD motif contribute to the interaction between Hsp40 or Hsp40‐like proteins and Hsp70. The HSFs respond to environmental stress by regulating gene expression. The transcriptional regulation of human *Hsp70* gene occurs through the activation of HSFs, and HSP70 can also be used as a negative regulator of HSFs (Abravaya et al., 1992). Our results supported the speculative model that the inactive state of HSFs is maintained by Hsp70–Hsp90 multipartite complexes under normal conditions. However, under stress, some denatured protein competes with HSF to bind to Hsp70, which activates HSFs and further increases the transcription and expression of *HSP70*. The increased Hsp70, in turn, inhibited the expression of HSFs. Our results provide the foundation for future wheat HSP studies for clarifying the interaction between HSPs and pathogen defense.

## 5 CONCLUSIONS

Heat‐shock proteins play a crucial role in development and responses to diverse stresses. Here, we systematically classified DnaJ (Hsp40) proteins into six groups according to the detailed structural characterization: HPD, DRD, DED, WAY, GK, and CR domains. Meanwhile, Hsp70 proteins were classified into four subfamilies according to the repetition of EXE motif in C‐terminal. Moreover, infection by *Bgt* and *Pst* triggered robust alteration in gene expression of Hsp‐encoding genes in wheat, but the expression profiles of these HSP‐encoding homologous genes varied in response to *Bgt* and *Pst*. The Y2H assay experiments showed that a direct interaction failed between TaDnaJ70, TaHsp70‐30b, and TaHsp90‐4b, but TaHsp70‐30b could interacted with HSF‐A9L directly and TaHsp90‐4b indirectly. These indicate that the Hsp protein‐encoding genes of wheat responded to *Bgt* and *Pst* stress and played important roles in responding to fungal stress by a more complex pathway than that in the model plant.

## AUTHORS’ CONTRIBUTIONS

HZ and WJ conceived the project and provided overall supervision of the study; HG, HZ and GW performed the experiments and data analysis; YW and CW contribution to developing the materials; XL helped in experimental works; HZ wrote the first version of the paper; all authors reviewed and approved the final manuscript.

## AVAILABILITY OF DATA AND MATERIALS

All data generated and analyzed during this study are included in this published article and its supplemental material. And the raw data of RNA‐Seq were deposited into NCBI under accession No. PRJNA243835

## COMPETING INTERESTS

The authors declare that they have no competing interests. The plant specimens used in our study are not an endangered species.

## FUNDING INFORMATION

This work was financially supported by the National Key Research and Development Program of China (grant no. 2017YFD0100701) and the Program of Introducing Talents of Innovative Discipline to Universities (Project 111, B18042).

## Supporting information

Supplemental Table S1. Details regarding primers used for the qRT‐PCR analysis and PCR amplification of HSP‐encoding genes
Supplemental Table S2. Wheat DnaJ proteins
Supplemental Table S3. Wheat 70‐kD heat‐shock proteins
Supplemental Table S4. Wheat 90‐kD heat‐shock proteins
Supplemental Figure S1. Expression profiles of the *TaDnaJ* genes in the three time periods of *Bgt* and *Pst* treatment
Supplemental Figure S2. Expression profiles of the *TaHSP70* and *TaHSP90* genes in the three time periods of *Bgt* and *Pst* treatment
Supplemental Figure S3. Expression patterns of differentially expressed *DnaJ* genes in N9134 infected by *Bgt* and *Pst*
Supplemental Figure S4. Expression patterns of differentially expressed *Hsp70* and *Hsp90* genes in N9134 infected by *Bgt* and *Pst*
Supplemental Figure S5. Relative expression of genes encoding *Bgt*‐HSP during the wheat–*Bgt* interaction
Supplemental Figure S6. Cytological observations of N9134R/S‐*Bgt* isolate E09 interaction

## References

[tpg220092-bib-0001] Abravaya, K. , Myers, M. P. , Murphy, S. P. , & Morimoto, R. I. (1992). The human heat‐shock protein Hsp70 interacts with HSF, the transcription factor that regulates heat‐shock gene‐expression. Genes & Development, 6, 1153–1164. 10.1101/gad.6.7.1153 1628823

[tpg220092-bib-0002] Ahmad, A. , Bhattacharya, A. , McDonald, R. A. , Cordes, M. , Ellington, B. , Bertelsen, E. B. , & Zuiderweg, E. R. (2011). Heat shock protein 70 kDa chaperone/DnaJ cochaperone complex employs an unusual dynamic interface. Proceedings of the National Academy of Sciences of the United States of America, 108, 18966–18971. 10.1073/pnas.1111220108 22065753 PMC3223468

[tpg220092-bib-0003] Alderson, T. R. , Kim, J. H. , & Markley, J. L. (2016). Dynamical structures of Hsp70 and Hsp70‐Hsp40 complexes. Structure, 24, 1014–1030. 10.1016/j.str.2016.05.011 27345933 PMC4938735

[tpg220092-bib-0004] Alvira, S. , Cuellar, J. , Rohl, A. , Yamamoto, S. , Itoh, H. , Alfonso, C. , Rivas, G. , Buchner, J. , & Valpuesta, J. M. (2014). Structural characterization of the substrate transfer mechanism in Hsp70/Hsp90 folding machinery mediated by Hop. Nature Communications, 5, 5484. 10.1038/ncomms6484 25407331

[tpg220092-bib-0005] Bailey, T. L. , Boden, M. , Buske, F. A. , Frith, M. , Grant, C. E. , Clementi, L. , Ren, J. , Li, W. W. , & Noble, W. S. (2009). MEME SUITE: Tools for motif discovery and searching. Nucleic Acids Research, 37, W202–W208. 10.1093/nar/gkp335 19458158 PMC2703892

[tpg220092-bib-0006] Benbahouche Nel, H. , Iliopoulos, I. , Torok, I. , Marhold, J. , Henri, J. , Kajava, A. V. , Farkaš, R. , Kempf, T. , Schnölzer, M. , Meyer, P. , Kiss, I. , Bertrand, E. , Mechler, B. M. , & Pradet‐Balade, B. (2014). *Drosophila* Spag is the homolog of RNA polymerase II‐associated protein 3 (RPAP3) and recruits the heat shock proteins 70 and 90 (Hsp70 and Hsp90) during the assembly of cellular machineries. Journal of Biological Chemistry, 289, 6236–6247. 10.1074/jbc.M113.499608 24394412 PMC3937688

[tpg220092-bib-0007] Bukau, B. , Weissman, J. , & Horwich, A. (2006). Molecular chaperones and protein quality control. Cell, 125, 443–451. 10.1016/j.cell.2006.04.014 16678092

[tpg220092-bib-0008] Cintron, N. S. , & Toft, D. (2006). Defining the requirements for Hsp40 and Hsp70 in the Hsp90 chaperone pathway. Journal of Biological Chemistry, 281, 26235–26244. 10.1074/jbc.M605417200 16854979

[tpg220092-bib-0009] Cuellar, J. , Perales‐Calvo, J. , Muga, A. , Valpuesta, J. M. , & Moro, F. (2013). Structural insights into the chaperone activity of the 40‐kDa heat shock protein DnaJ: Binding and remodeling of a native substrate. Journal of Biological Chemistry, 288, 15065–15074. 10.1074/jbc.M112.430595 23580641 PMC3663527

[tpg220092-bib-0010] Cui, Y. , Zhang, X. , Gong, Y. , Niu, S. , Yin, N. , Yao, R. , Xu, W. , Li, D. , Wang, H. , He, Y. , Cao , J. , & Yin, Y. (2011). Immunization with DnaJ (hsp40) could elicit protection against nasopharyngeal colonization and invasive infection caused by different strains of *Streptococcus pneumoniae* . Vaccine, 29, 1736–1744. 10.1016/j.vaccine.2010.12.126 21238570

[tpg220092-bib-0011] de la Fuente van Bentem, S. , Vossen, J. H. , de Vries, K. J. , van Wees, S. , Tameling, W. I. , Dekker, H. L. , de Koster, C. G. , Haring, M. A. , Takken, F. L. W. , & Cornelissen, B. J. (2005). Heat shock protein 90 and its co‐chaperone protein phosphatase 5 interact with distinct regions of the tomato I‐2 disease resistance protein. Plant Journal, 43, 284–298. 10.1111/j.1365-313X.2005.02450.x 15998314

[tpg220092-bib-0012] Duan, Y. H. , Guo, J. , Ding, K. , Wang, S. J. , Zhang, H. , Dai, X. W. , Chen, Y. Y. , Govers, F. , Huang, L. L. , & Kang, Z. S. (2011). Characterization of a wheat HSP70 gene and its expression in response to stripe rust infection and abiotic stresses. Molecular Biology Reports, 38, 301–307. 10.1007/s11033-010-0108-0 20349142

[tpg220092-bib-0013] Fiaux, J. , Horst, J. , Scior, A. , Preissler, S. , Koplin, A. , Bukau, B. , & Deuerling, E. (2010). Structural analysis of the ribosome‐associated complex (RAC) reveals an unusual Hsp70/Hsp40 interaction. Journal of Biological Chemistry, 285, 3227–3234. 10.1074/jbc.M109.075804 19920147 PMC2823442

[tpg220092-bib-0014] Fu, Y. , Zhang, H. , Mandal, S. N. , Wang, C. , Chen, C. , & Ji, W. (2016). Quantitative proteomics reveals the central changes of wheat in response to powdery mildew. Journal of Proteomics, 130, 108–119. 10.1016/j.jprot.2015.09.006 26381202

[tpg220092-bib-0015] Hennessy, F. , Nicoll, W. S. , Zimmermann, R. , Cheetham, M. E. , & Blatch, G. L. (2005). Not all J domains are created equal: Implications for the specificity of Hsp40‐Hsp70 interactions. Protein Science, 14, 1697–1709. 10.1110/ps.051406805 15987899 PMC2253343

[tpg220092-bib-0016] Hu, W. G. , Wang, Q. H. , Wang, S. W. , Wang, M. M. , Wang, C. Y. , Tian, Z. R. , Liu, X. , Ji, W. , & Zhang, H. (2020). Gene co‐expression network analysis provides a novel insight into the dynamic response of wheat to powdery mildew stress. Journal of Genetics, 99, 44. 10.1007/s12041-020-01206-w 32529987

[tpg220092-bib-0017] Kadota, Y. , & Shirasu, K. (2012). The HSP90 complex of plants. Biochimica et Biophysica Acta, 1823, 689–697. 10.1016/j.bbamcr.2011.09.016 22001401

[tpg220092-bib-0018] Kadota, Y. , Shirasu, K. , & Guerois, R. (2010). NLR sensors meet at the SGT1‐HSP90 crossroad. Trends in Biochemical Sciences, 35, 199–207. 10.1016/j.tibs.2009.12.005 20096590

[tpg220092-bib-0019] Kampinga, H. H. , & Craig, E. A. (2010). The HSP70 chaperone machinery: J proteins as drivers of functional specificity. Nature Reviews: Molecular Cell Biology, 11, 579–592. 10.1038/nrm2941 20651708 PMC3003299

[tpg220092-bib-0020] Kanzaki, H. , Saitoh, H. , Ito, A. , Fujisawa, S. , Kamoun, S. , Katou, S. , Yoshioka H. , & Terauchi, R. (2003). Cytosolic HSP90 and HSP70 are essential components of INF1‐mediated hypersensitive response and non‐host resistance to *Pseudomonas cichorii* in *Nicotiana benthamiana* . Molecular Plant Pathology, 4, 383–391. 10.1046/j.1364-3703.2003.00186.x 20569398

[tpg220092-bib-0021] Landi, S. , Capasso, G. , Ben Azaiez, F. E. , Jallouli, S. , Ayadi, S. , Trifa, Y. , & Esposito, S. (2019). Different roles of heat shock proteins (70 kDa) during abiotic stresses in barley (*Hordeum vulgare*) genotypes. Plants, 8, 248. 10.3390/plants8080248 31357401 PMC6724185

[tpg220092-bib-0022] Leng, L. , Liang, Q. , Jiang, J. , Zhang, C. , Hao, Y. , Wang, X. , & Su, W. (2017). A subclass of HSP70s regulate development and abiotic stress responses in *Arabidopsis thaliana* . Journal of Plant Research, 130, 349–363. 10.1007/s10265-016-0900-6 28004282

[tpg220092-bib-0023] Liu, J. Z. , & Whitham, S. A. (2013). Overexpression of a soybean nuclear localized type‐III DnaJ domain‐containing HSP40 reveals its roles in cell death and disease resistance. Plant Journal, 74, 110–121. 10.1111/tpj.12108 23289813

[tpg220092-bib-0024] Murphy, M. E. (2013). The HSP70 family and cancer. Carcinogenesis, 34, 1181–1188. 10.1093/carcin/bgt111 23563090 PMC3670260

[tpg220092-bib-0025] Nejat, N. , & Mantri, N. (2017). Plant immune system: Crosstalk between responses to biotic and abiotic stresses the missing link in understanding plant defence. Current Issues in Molecular Biology, 23, 1–16. 10.21775/cimb.023.001 28154243

[tpg220092-bib-0026] Nollen, E. A. A. , & Morimoto, R. I. (2002). Chaperoning signaling pathways: Molecular chaperones as stress‐sensing ‘heat shock’ proteins. Journal of Cell Science, 115, 2809–2816. 12082142 10.1242/jcs.115.14.2809

[tpg220092-bib-0027] Papsdorf, K. , & Richter, K. (2014). Protein folding, misfolding and quality control: The role of molecular chaperones. Essays in Biochemistry, 56, 53–68. 10.1042/bse0560053 25131586

[tpg220092-bib-0028] Pearl, L. H. , & Prodromou, C. (2006). Structure and mechanism of the Hsp90 molecular chaperone machinery. Annual Review of Biochemistry, 75, 271–294. 10.1146/annurev.biochem.75.103004.142738 16756493

[tpg220092-bib-0029] Qiu, X. B. , Shao, Y. M. , Miao, S. , & Wang, L. (2006). The diversity of the DnaJ/Hsp40 family, the crucial partners for Hsp70 chaperones. Cellular and Molecular Life Sciences, 63, 2560–2570. 10.1007/s00018-006-6192-6 16952052 PMC11136209

[tpg220092-bib-0030] Ranek, M. J. , Stachowski, M. J. , Kirk, J. A. , & Willis, M. S. (2018). The role of heat shock proteins and co‐chaperones in heart failure. Philosophical Transactions of the Royal Society of London. Series B: Biological Sciences, 373, 20160530. 10.1098/rstb.2016.053020160530 29203715 PMC5717530

[tpg220092-bib-0031] Saijo, Y. , Loo, E. P. , & Yasuda, S. (2018). Pattern recognition receptors and signaling in plant‐microbe interactions. Plant Journal, 93, 592–613. 10.1111/tpj.13808 29266555

[tpg220092-bib-0032] Shimizu, T. , Yoshii, A. , Sakurai, K. , Hamada, K. , Yamaji, Y. , Suzuki, M. , Namba, S. , & Hibi, T. (2009). Identification of a novel tobacco DnaJ‐like protein that interacts with the movement protein of tobacco mosaic virus. Archives Virology, 154, 959–967. 10.1007/s00705-009-0397-6 19458900

[tpg220092-bib-0033] Sung, D. Y. , Kaplan, F. , & Guy, C. L. (2001). Plant Hsp70 molecular chaperones: Protein structure, gene family, expression and function. Physiologia Plantarum, 113, 443–451. 10.1034/j.1399-3054.2001.1130402.x

[tpg220092-bib-0034] Sung, Y. Y. , Rahman, N. A. , Shazili, N. A. M. , Chen, S. J. , Lv, A. J. , Sun, J. F. , Shi H. , & MacRae, T. H. (2018). Non‐lethal heat shock induces Hsp70 synthesis and promotes tolerance against heat, ammonia and metals in post‐larvae of the white leg shrimp *Penaeus vannamei* (Boone, 1931). Aquaculture, 483, 21–26. 10.1016/j.aquaculture.2017.09.034

[tpg220092-bib-0035] Szabo, A. , Korszun, R. , Hartl, F. U. , & Flanagan, J. (1996). A zinc finger‐like domain of the molecular chaperone DnaJ is involved in binding to denatured protein substrates. EMBO Journal, 15, 408–417. 10.1002/j.1460-2075.1996.tb00371.x 8617216 PMC449956

[tpg220092-bib-0036] Takahashi, A. , Casais, C. , Ichimura, K. , & Shirasu, K. (2003). HSP90 interacts with RAR1 and SGT1 and is essential for RPS2‐mediated disease resistance in Arabidopsis. PNAS, 100, 11777–11782. 10.1073/pnas.2033934100 14504384 PMC208834

[tpg220092-bib-0037] Tamura, K. , Stecher, G. , Peterson, D. , Filipski, A. , & Kumar, S. (2013). MEGA6: Molecular evolutionary genetics analysis version 6.0. Molecular Biology and Evolution, 30, 2725–2729. 10.1093/molbev/mst197 24132122 PMC3840312

[tpg220092-bib-0038] Wallin, R. P. A. , Lundqvist, A. , More, S. H. , von Bonin, A. , Kiessling, R. , & Ljunggren, H. G. (2002). Heat‐shock proteins as activators of the innate immune system. Trends in Immunology, 23, 130–135. 10.1016/S1471-4906(01)02168-8 11864840

[tpg220092-bib-0039] Wang, G. F. , Wei, X. , Fan, R. , Zhou, H. , Wang, X. , Yu, C. , Dong, L. , Dong, Z. , Wang, X. , Kang, Z. , Ling, H. , Shen, Q. H. , Wang, D. , & Zhang, X. (2011). Molecular analysis of common wheat genes encoding three types of cytosolic heat shock protein 90 (Hsp90): Functional involvement of cytosolic Hsp90s in the control of wheat seedling growth and disease resistance. New Phytologist, 191, 418–431. 10.1111/j.1469-8137.2011.03715.x 21488877

[tpg220092-bib-0040] Wen, F. , Wu, X. , Li, T. , Jia, M. , Liu, X. , Li, P. , Zhou, X. , Ji, X. , & Yue, X. (2017). Genome‐wide survey of heat shock factors and heat shock protein 70s and their regulatory network under abiotic stresses in *Brachypodium distachyon* . PLoS ONE, 12, e0180352. 10.1371/journal.pone.0180352 28683139 PMC5500289

[tpg220092-bib-0041] Weyer, F. A. , Gumiero, A. , Gese, G. V. , Lapouge, K. , & Sinning, I. (2017). Structural insights into a unique Hsp70‐Hsp40 interaction in the eukaryotic ribosome‐associated complex. Nature Structural & Molecular Biology, 24, 144–151. 10.1038/nsmb.3349 28067917

[tpg220092-bib-0042] Xue, F. , Ji, W. , Wang, C. , Zhang, H. , & Yang, B. (2012). High‐density mapping and marker development for the powdery mildew resistance gene PmAS846 derived from wild emmer wheat (*Triticum turg*idum var. *dicoccoides*). Theoretical and Applied Genetics, 124, 1549–1560. 10.1007/s00122-012-1809-7 22350087

[tpg220092-bib-0043] Yer, E. N. , Baloglu, M. C. , & Ayan, S. (2018). Identification and expression profiling of all Hsp family member genes under salinity stress in different poplar clones. Gene, 678, 324–336. 10.1016/j.gene.2018.08.049 30110648

[tpg220092-bib-0044] Zarouchlioti, C. , Parfitt, D. A. , Li, W. , Gittings, L. M. , & Cheetham, M. E. (2017). DNAJ Proteins in neurodegeneration: Essential and protective factors. Philosophical Transactions of the Royal Society B: Biological Sciences, 373, 20160534. 10.1098/rstb.2016.0534 PMC5717533 29203718

[tpg220092-bib-0045] Zhang, H. , Fu, Y. , Guo, H. , Zhang, L. , Wang, C. , Song, W. , Yan, Z. , Wang, Y. , & Ji, W. (2019a). Transcriptome and proteome‐based network analysis reveals a model of gene activation in wheat resistance to stripe rust. International Journal of Molecular Sciences, 20, 1106. 10.3390/ijms20051106 30836695 PMC6429138

[tpg220092-bib-0046] Zhang, H. , Mao, R. , Wang, Y. , Zhang, L. , Wang, C. , Lv, S. , Liu, X. , Wang, Y. , & Ji, W. (2019b). Transcriptome‐wide alternative splicing modulation during plant‐pathogen interactions in wheat. Plant Science, 288, 110160. 10.1016/j.plantsci.2019.05.023 31521219

[tpg220092-bib-0047] Zhang, H. , Yang, Y. , Wang, C. , Liu, M. , Li, H. , Fu, Y. , Wang, Y. , Nie, Y. , Liu, X. , & Ji, W. (2014). Large‐scale transcriptome comparison reveals distinct gene activations in wheat responding to stripe rust and powdery mildew. BMC Genomics, 15, 898. 10.1186/1471-2164-15-898 25318379 PMC4201691

[tpg220092-bib-0048] Zhong, X. , Yang, J. , Shi, Y. , Wang, X. , & Wang, G. L. (2018). The DnaJ protein OsDjA6 negatively regulates rice innate immunity to the blast fungus *Magnaporthe oryzae* . Molecular Plant Pathology, 19, 607–614. 10.1111/mpp.12546 28220688 PMC6638105

[tpg220092-bib-0049] Zuehlke, A. D. , Moses, M. A. , & Neckers, L. (2017). Heat shock protein 90: Its inhibition and function. Philosophical Transactions of the Royal Society B: Biological Sciences, 373, 20160527. 10.1098/rstb.2016.0527 PMC5717527 29203712

