# Supplementary material for: Identification and expression analysis of heat‐shock proteins in wheat infected with powdery mildew and stripe rust
Source: Plant Genome. 2021 Mar 14;14(2):e20092. doi: 10.1002/tpg2.20092 (PMC12807163; doi:10.1002/tpg2.20092)
Supplement: Supplementary file 1 [file TPG2-14-e20092-s001.pdf]

Table S1. Details regarding primers used for the qRT-PCR analysis and PCR amplification of HSP-encoding genes.

Primer	Forward primer sequence	Reverse primer sequence
qTaHsf-B1	5- CTTCACGGAATGGAGCAACG -3	5- GGCGGCTACTATTCTTCGTTTG -3
qTaDNAJ70b	5- TGAGTGACCCTGAGAAGCGTGAG -3	5- CACCACCTCCTCCACCCATT -3
qTaHsp70-30b	5- CAAGGGTGAGGACAAGCAGT -3	5- CCTCTGGGAATCATTGAAGTAA -3
qTaHsp90-4b	5- CAAGTCGGACCTCGTCAACA -3	5- GAAACCGACACCAAACTGCC -3
BD-TaDNAJ70b	5-CATGGAGGCCGAATTCATGTTCGGCCGCGCCC-3	5-CTGCAGGTCGACGGATTTACTGTTGTGCGCAC-3
AD-TaHsp70-30b	5-GGAGGCCAGTGAATTCATGGCGCCGACCAAGG-3	5-CTCGAGCTCGATGGATTTAGTCGACCTCCTCGA-3
AD-TaHsp90-4b	5-GGAGGCCAGTGAATTCATGGCGACGGAGACCGAGA-3	5-CTCGAGCTCGATGGATTTAGTCGACCTCCTCCA-3
BD-TaHsp70-30b	5-CATGGAGGCCGAATTCATGGCGCCGACCAAGG-3	5-CTGCAGGTCGACGGATTTAGTCGACCTCCTCGA-3
AD-DNAJ70b-JD	5-GGAGGCCAGTGAATTCATGTACTACGAGGTGCTCG-3	5-CTCGAGCTCGATGGATTTAATCATATATCTCACGC-3
AD-DNAJ70b-JDC	5-GGAGGCCAGTGAATTCATGTACTACGAGGTGCTCG-3	5-CTCGAGCTCGATGGATTTACTCCTGAAGAACCTTC-3
AD-DNAJ70b-CT	5-GGAGGCCAGTGAATTCATGCTTCAGGAGAAGAAAG-3	5-CTCGAGCTCGATGGATTTACAGCGAGTCGGGGAAC-3

Table S2. Wheat DnaJ proteins

Number	Domains	Type	Gene ID in chromosome A	Gene ID in chromosome B	Gene ID in chromosome D	paralogue protein loci
**TaDNAJ 2**	J+DRD+CT	Ia			TraesCS1D02G004800	
**TaDNAJ 3**	AR+DD+J	Vb	TraesCS1A02G020600	TraesCS1B02G024600	TraesCS1D02G020100	
**TaDNAJ 4**	J+DRD+2C+WAY	Va	TraesCS1A02G042500	TraesCS1B02G054900	TraesCS1D02G042900	
**TaDNAJ 5**	J+DRD+GF	IIb	TraesCS1A02G070300	TraesCS1B02G088700	TraesCS1D02G072800	
**TaDNAJ 7**	J+DRD+GF+CT	IIb	TraesCS1A02G099300[P]	TraesCS1B02G125100[P,S]	TraesCS1D02G107200[P]	
**TaDNAJ 13**	J+DRD+CT	III	TraesCS1A02G175900	TraesCS1B02G204300	TraesCS1D02G162900	
**TaDNAJ 9**	NT+J+LR+AS	Vb	TraesCS1A02G193300	TraesCS1B02G208100	TraesCS1D02G197000	
**TaDNAJ 12**	J+DRD+AR+CT	III	TraesCS1A02G223900	TraesCS1B02G237200	TraesCS1D02G225500	
**TaDNAJ 49**	NT+J+DRD+CT	IIa	TraesCS1A02G230000	TraesCS1B02G244100	TraesCS1D02G232000	
**TaDNAJ 14**	GF+AP+J+DRD+CT	VI	TraesCS1A02G234900[P]	TraesCS1B02G248800[P]	TraesCS1D02G237300[P]	
**TaDNAJ 16**	AP+J+CT	III	TraesCS1A02G248900	TraesCS1B02G259600	TraesCS1D02G248400	
**TaDNAJ 17**	AP+DD+J	VI	TraesCS1A02G251500	TraesCS1B02G262100	TraesCS1D02G251000	
**TaDNAJ 18**	GF+J+DRD+GR+CT	IIb	TraesCS1A02G267600	TraesCS1B02G278300	TraesCS1D02G267500	
**TaDNAJ 19**	AP+J+DRD+CT	III	TraesCS1A02G349900	TraesCS1B02G364200	TraesCS1D02G352600	
**TaDNAJ 10**	J+DRD+G/F	Ib	TraesCS1A02G366400	TraesCS1B02G384100	TraesCS1D02G372000	
**TaDNAJ 21**	J+DRD+GF+CT	IIb	TraesCS1A02G395300	TraesCS1B02G423600	TraesCS1D02G403500[P]	
**TaDNAJ 22**	AP+Un+J	VI	TraesCS1A02G419500	TraesCS1B02G449600	TraesCS1D02G427300	
**TaDNAJ 23**	GR+J+DRD+CT	Vb	TraesCS1A02G420500[P]	TraesCS1B02G451500	TraesCS1D02G428400[P]	
**TaDNAJ 24**	J+DRD+DD	III	TraesCS2A02G150400	TraesCS2B02G175600	TraesCS2D02G155500	TraesCS6A02G059000LC
**TaDNAJ 25**	NT+J+DRD+CT	III	TraesCS2A02G160100	TraesCS2B02G186400	TraesCS2D02G167500	
**TaDNAJ 26**	AP+J+DRD+CT	III	TraesCS2A02G272700	TraesCS2B02G290900	TraesCS2D02G272000	
**TaDNAJ 27**	J+DRD+AP+2C+DD+CT	Va	TraesCS2A02G284200	TraesCS2B02G301100	TraesCS2D02G283100	
**TaDNAJ 28**	SP+J+DRD+CT	Va	TraesCS2A02G498100	TraesCS2B02G526300	TraesCS2D02G498200	
**TaDNAJ 29**	SP+J+DRD+AP+CT	III	TraesCS2A02G556100	TraesCS2B02G586400	TraesCS2D02G557300	
**TaDNAJ 30**	NT+DD+J	VI	TraesCS2A02G576600	TraesCS2B02G610800	TraesCS2D02G587300	
**TaDNAJ 31**	DD+J+DRD+CT	Ia		TraesCS3B02G004700	TraesCS3D02G008500	TraesCSU02G039400[P]
**TaDNAJ 20**	AP+J+DRD+CT	Ia	TraesCS3A02G083700	TraesCS3B02G098700		
**TaDNAJ 32**	J+DRD+GF+CT	IIb	TraesCS3A02G161200	TraesCS3B02G192300	TraesCS3D02G168500	
**TaDNAJ 33**	2GR+GF+J	VI	TraesCS3A02G187500[P]	TraesCS3B02G216900[P]	TraesCS3D02G191200[P]	
**TaDNAJ 34**	J+DRD+GS	III			TraesCS3D02G193500	

**TaDNAJ 72**	KH+GF+J+DRD+CT	III	TraesCS3A02G190100		TraesCS3D02G193600	
**TaDNAJ 35**	AP+J+DRD+CT	IIa	TraesCS3A02G201300	TraesCS3B02G225000	TraesCS3D02G203800	
**TaDNAJ 15**	J+DRD+CT	Ia	TraesCS3A02G202200		TraesCS3D02G201400	
**TaDNAJ 36**	J+DRD+2C+DD+GF+DD+CT	Va	TraesCS3A02G213800[P]	TraesCS3B02G244000	TraesCS3D02G215900[P]	
**TaDNAJ 37**	J+DRD+CT	Ia	TraesCS3A02G216800	TraesCS3B02G247200	TraesCS3D02G218800	
**TaDNAJ 38**	J+DRD+CT	Ib	TraesCS3A02G254700	TraesCS3B02G286700	TraesCS3D02G255600	
**TaDNAJ 39**	AR+J+DRD+CT	III	TraesCS3A02G282100	TraesCS3B02G315800	TraesCS3D02G282100	
**TaDNAJ 40**	NT+J+CT	VI	TraesCS3A02G309700[S]	TraesCS3B02G152700	TraesCS3D02G135400	
**TaDNAJ 41**	J+DRD+GF+CT	IIb	TraesCS3A02G386400	TraesCS3B02G418500	TraesCS3D02G379400	
**TaDNAJ 42**	J+DRD+2C+RR+DD+CT	Va	TraesCS3A02G435100	TraesCS3B02G470100	TraesCS3D02G518300LC	
**TaDNAJ 43**	J+DRD+CT	IIb	TraesCS3A02G520100	TraesCS3B02G587800	TraesCSU02G059600	
**TaDNAJ 44**	J+DRD+2C+2DD+CT	Va	TraesCS3A02G536400[P]	TraesCS3B02G607500	TraesCS3D02G541800	
**TaDNAJ 45**	LK+J+DRD+GF	VI	TraesCS3A02G537600[P]	TraesCS3B02G603100[P]	TraesCS3D02G543100[P]	
**TaDNAJ 46**	GR+J+DRD+CT	Vb	TraesCS4A02G521400LC			
**TaDNAJ 47**	AR+J+AS+DD	Vb	TraesCS4A02G353900			TraesCS5B02G518600
**TaDNAJ 48**	NT+J	VI	TraesCS4A02G256200	TraesCS4B02G058500	TraesCS4D02G058400	
**TaDNAJ 50**	NT+J+DRD+2C+DD+DD	Va	TraesCS4A02G214500	TraesCS4B02G101500	TraesCS4D02G095600LC	
**TaDNAJ 51**	AS+J+DRD+CT	VI	TraesCS4A02G203300	TraesCS4B02G107200	TraesCS4D02G104100	
**TaDNAJ 52**	NT+J+DRD+CT	Ia		TraesCS4B02G175400	TraesCS4D02G177300	
**TaDNAJ 53**	AS+J+DRD+CT	Ia	TraesCS4A02G116800	TraesCS4B02G187600	TraesCS4D02G189000	
**TaDNAJ 54**	NT+J+DRD+GF+CT	Ia	TraesCS4A02G110600	TraesCS4B02G193500	TraesCS4D02G194500	
**TaDNAJ 55**	NT+J+DRD+CT	III	TraesCS4A02G087200[P]	TraesCS4B02G217200[P]	TraesCS4D02G217500	
**TaDNAJ 11**	SP+J+DRD+RR	Ia	TraesCS4A02G069100	TraesCS4B02G226300	TraesCS4D02G226900	
**TaDNAJ 56**	SP+J+DRD+GF+4CC+CT	IIa	TraesCS4A02G064300	TraesCS4B02G241700	TraesCS4D02G241300	
**TaDNAJ 57**	GS+J	VI	TraesCS4A02G041000	TraesCS4B02G261900	TraesCS4D02G261900	
**TaDNAJ 58**	J+DRD+AP+2C+DD+J	Va		TraesCS4B02G319100[P]	TraesCS4D02G315600[P]	TraesCS5A02G493100
**TaDNAJ 59**	J+DRD+AR+CT	III		TraesCS4B02G334200		
**TaDNAJ 60**	J+DRD+CT	Ia			TraesCS4D02G334100	
**TaDNAJ 61**	AS+J+DRD+CT	IIa	TraesCS5A02G045300[P]	TraesCS5B02G049500	TraesCS5D02G055400[P]	
**TaDNAJ 62**	J+DRD+CT	Ia	TraesCS5A02G061200[P,S]	TraesCS5B02G070900[P,S]	TraesCS5D02G072700[P,S]	
**TaDNAJ 63**	J+DRD+GF+CT	VI	TraesCS5A02G106000[P,S]	TraesCS5B02G114500[P,S]	TraesCS5D02G116700[P,S]	
**TaDNAJ 64**	SP+J+DRD+GF+4CC+CT	IIa	TraesCS5A02G115000	TraesCS5B02G115900	TraesCS5D02G125500	

**TaDNAJ 65**	SP+J+DRD+GF+4CC+CT	IIa	TraesCS5A02G117600		TraesCS5D02G129700	
**TaDNAJ 66**	GR+J+DRD+CT	Ib	TraesCS5A02G190500	TraesCS5B02G192700	TraesCS5D02G200400	
**TaDNAJ 67**	AS+J+DRD+CT	Ib	TraesCS5A02G190700	TraesCS5B02G192500	TraesCS5D02G200200	
**TaDNAJ 68**	J+DRD+CT	Ia	TraesCS5A02G240700			
**TaDNAJ 69**	J+DRD+AR+CT	Ia	TraesCS5A02G271700	TraesCS5B02G272000	TraesCS5D02G279400	
**TaDNAJ 70**	J+DRD+GF+4CC+CT	IIa	TraesCS5A02G372900[P,S]	TraesCS5B02G374900	TraesCS5D02G382400[P,S]	
**TaDNAJ 71**	DD+J	VI	TraesCS5A02G391600	TraesCS5B02G396500	TraesCS5D02G401400	
**TaDNAJ 73**	AP+J+DRD+CT	Ia	TraesCS5A02G399900	TraesCS5B02G404700	TraesCS5D02G409800	
**TaDNAJ 74**	J+DRD+GF+4CC+CT	IIa	TraesCS5A02G426100	TraesCS5B02G428000	TraesCS5D02G434100	
**TaDNAJ 75-1**	AP+J+DRD+CT	III		TraesCS5B02G496700		
**TaDNAJ 75-2**	AP+J+CT	III		TraesCS5B02G496800	5DL:437178_AA1465520	
**TaDNAJ 76**	AP+J+DRD+CT	III	TraesCS5A02G483600	TraesCS5B02G496900	TraesCS5D02G497200	
**TaDNAJ 77**	GR+J	VI	TraesCS5A02G559800LC	TraesCS5B02G607100LC[P]	TraesCS5D02G420700[P]	
**TaDNAJ 78**	AP+J+DRD+CT	III		TraesCS5B02G508500	TraesCS5D02G508400	TraesCS4A02G365000
**TaDNAJ 79**	SP+J+DRD+CT	IIa		TraesCS5B02G511600	TraesCS5D02G512100	TraesCS4A02G361300
**TaDNAJ 80**	AP+J+GR+CT	Va		TraesCS5B02G519100		
**TaDNAJ 81**	AR+J+AP+CT	Vb			TraesCS5D02G518400	
**TaDNAJ 82**	J+DRD+SP+CT	IIb	TraesCS6A02G087000	TraesCS6B02G115000		TraesCSU02G019500
**TaDNAJ 83**	J+DRD+CT	III		TraesCS6B02G114100	TraesCS6D02G076700	
**TaDNAJ 84**	J+DRD+CT	III		6BS:514431_AA1659570		U:645311_AA2143950
**TaDNAJ 85**	J+DRD+CT	IIIb	TraesCS6A02G103600LC			
**TaDNAJ 6**	NT+J+DRD+CT	Ia	TraesCS6A02G158600	TraesCS6B02G192700	TraesCS6D02G153900	
**TaDNAJ 86**	NT+J+SP+2C+DD+CT	Va	TraesCS6A02G167100[P]	TraesCS6B02G194500[P]	TraesCS6D02G155600[P]	
**TaDNAJ 87**	J+DRD+GF+4CC+CT	IIb	TraesCS6A02G250800	TraesCS6B02G274600	TraesCS6D02G232600	
**TaDNAJ 88**	GR+J+DRD+CT	Ia	TraesCS6A02G264600	TraesCS6B02G546300LC	TraesCS6D02G250300	
**TaDNAJ 89**	AS+J+DRD+CT	Ia	TraesCS6A02G310200	TraesCS6B02G340100	TraesCS6D02G289600	
**TaDNAJ 90**	NT+J+DRD+CT	Ia	TraesCS6A02G314500	TraesCS6B02G344500	TraesCS6D02G293800	
**TaDNAJ 91**	SP+J+DRD+CT	Ia	TraesCS6A02G341900	TraesCS6B02G373800	TraesCS6D02G322200	
**TaDNAJ 92**	AP+J+DRD+GF+4CC+CT	IIa	TraesCS6A02G372700	TraesCS6B02G410600	TraesCS6D02G356800	
**TaDNAJ 93**	J+DRD+GR+CT	IIb	TraesCS6A02G421300			
**TaDNAJ 94**	ASP+J+DRD+GF+4CC+CT	IIa	TraesCS7A02G051000			
**TaDNAJ 95**	J+DRD+GF+CT	Ib	TraesCS7A02G159500	TraesCS7B02G064000		

**TaDNAJ 96**	J+DRD+GF+CT	Ia	TraesCS7A02G159600	TraesCS7B02G064100	
**TaDNAJ 97**	J+DRD+GF+CT	Ia			TraesCS7D02G160400
**TaDNAJ 1**	SP+J+DRD+GF+4CC+CT	IIa	TraesCS7A02G176600	TraesCS7B02G081600	TraesCS7D02G177900
**TaDNAJ 98**	AP+J+DRD+CT	Ia	TraesCS7A02G194600	TraesCS7B02G100200	TraesCS7D02G196200
**TaDNAJ 99**	NT+J+DRD+CT	VI	TraesCS7A02G213900	TraesCS7B02G121200	TraesCS7D02G216000
**TaDNAJ 100**	AP+J+DRD+RR	Ia	TraesCS7A02G220300	TraesCS7B02G127100	TraesCS7D02G221800
**TaDNAJ 101**	NT+J+DRD+GF+CT	III	TraesCS7A02G235100	TraesCS7B02G133300	TraesCS7D02G235100
**TaDNAJ 102**	J+DRD+2G/F	Ia	TraesCS7A02G250300	TraesCS7B02G140900	TraesCS7D02G249000
**TaDNAJ 103**	SP+J+DRD+RR	Ia	TraesCS7A02G271600	TraesCS7B02G170400	TraesCS7D02G271900
**TaDNAJ 104**	J+DRD+GF+CT	IIb	TraesCS7A02G298000[P]	TraesCS7B02G188900	TraesCS7D02G293900
**TaDNAJ 105**	GR+J+DRD+CT	Ib	TraesCS7A02G305000	TraesCS7B02G205400	TraesCS7D02G300500
**TaDNAJ 106**	SP+J+GF+DED	IV	TraesCS7A02G506000	TraesCS7B02G694400LC	TraesCS7D02G494000
**TaDNAJ 107**	GF+J+GF+DED	IV	TraesCS7A02G506100		TraesCS7D02G494100
**TaDNAJ 8**	AS+J+DED+CT	IV	TraesCS7A02G506700		TraesCS7D02G494800
**TaDNAJ 108**	SP+GR+J+DED	IV			TraesCS7D02G494900
**TaDNAJ 109**	GS+J+DED	IV			TraesCS7D02G495200
**TaDNAJ 110**	SP+J+GF+DED	IV			TraesCS7D02G495500
**TaDNAJ 111**	GR+J+DED	IV		TraesCS7B02G413400	
**TaDNAJ 112**	NT+GF+J+DED+CT	IV		TraesCS7B02G414800	
**TaDNAJ 113**	AP+J+DED	IV		TraesCS7B02G415000	
**TaDNAJ 114-1**	GR+J+DRD+RR	Ia		TraesCS7B02G445500	TraesCS7D02G516600
**TaDNAJ 114-2**	GR+J+DRD+RR	Ia	TraesCS7A02G528700	TraesCS7B02G445600	TraesCS7D02G516700
**TaDNAJ 114-3**	GR+J+DRD+RR	Ia	TraesCS7A02G528800		TraesCS7D02G516800
**TaDNAJ 115**	AP+J+DRD+RR	Ia		7BL:577619_AA1879700	
**TaDNAJ 114-4**	GR+J+DRD+AR	Ia	TraesCS7A02G528900	TraesCS7B02G445700	TraesCS7D02G516900
**TaDNAJ 114-5**	GR+J+DRD+AR	Ia	TraesCS7A02G529000	TraesCS7B02G445800	TraesCS7D02G517000
**TaDNAJ 116**	NT+J+DRD+GR+CT	Ia		TraesCS7B02G445900	
**TaDNAJ 117-1**	GR+J+DRD+RR	Ia	TraesCS7A02G529400	TraesCS7B02G446000	
**TaDNAJ 117-2**	GR+J+DRD+RR	Ia		TraesCS7B02G446100	
**TaDNAJ 117-3**	GR+J+DRD+RR	Ia		TraesCS7B02G448500	
**TaDNAJ 118**	AP+J+DRD+CT	Ia		TraesCS7B02G498100	
**TaDNAJ 119**	SP+J+DED	IV		TraesCSU02G098500	

**TaDNAJ-CR 1**	GR+4CC	CR	TraesCS1A02G015100	TraesCS1B02G019300	1DS:080446_AA0248140
**TaDNAJ-CR 2**	AP+2CC	CR	TraesCS1A02G030000	TraesCS1B02G035300LC	TraesCS1D02G030500
**TaDNAJ-CR 3**	GR+4CC	CR	TraesCS2A02G058700	TraesCS2B02G070700	TraesCS2D02G057900
**TaDNAJ-CR 4**	SP+4CC	CR	TraesCS2A02G136700	TraesCS2B02G160600	TraesCS2D02G139400
**TaDNAJ-CR 5**	SP+4CC	CR	TraesCS2A02G136800		TraesCS2D02G139500
**TaDNAJ-CR 6**	SP+PA+4CC	CR	TraesCS2A02G384200	TraesCS2B02G401400	TraesCS2D02G380900
**TaDNAJ-CR 7**	AP+4CC	CR	TraesCS2A02G451100	TraesCS2B02G472600	TraesCS2D02G450800
**TaDNAJ-CR 8**	GF+4CC	CR	TraesCS3A02G219600	TraesCS3B02G250100	TraesCS3D02G232900
**TaDNAJ-CR 9**	RR+2CC	CR	TraesCS4A02G080900	TraesCS4B02G223100	TraesCS4D02G223500
**TaDNAJ-CR 10**	GR+4CC	CR	TraesCS4A02G298500	TraesCS4B02G015000	TraesCS4D02G013200
**TaDNAJ-CR 11**	SP+2CC	CR	TraesCS5A02G221700	TraesCS5B02G220500	TraesCS5D02G229500
**TaDNAJ-CR 12**	AP+4CC	CR	TraesCS6A02G197700	TraesCS6B02G218700	TraesCS6D02G183000
**TaDNAJ-CR 13**	AP+4CC	CR	TraesCS7A02G341000	TraesCS7B02G242200	TraesCS7D02G338600
**TaDNAJL1**	JL+DRD+AR+CT	JL	TraesCS1A02G393800		
**TaDNAJL2-1**	GF+JL	JL	TraesCS1A02G419000[S]	TraesCS1B02G449200[S]	TraesCS1D02G426800[S]
**TaDNAJL2-2**	GF+JL	JL	TraesCS1A02G419100[S]		
**TaDNAJL2-3**	GF+JL	JL	TraesCS1A02G419200[S]		
**TaDNAJL3**	LA+JL+CT	JL	TraesCS2A02G095200	TraesCS2B02G110300	TraesCS2D02G093500
**TaDNAJL4**	LA+JL+CT	JL	TraesCS2A02G155700	TraesCS2B02G180900	TraesCS2D02G161500
**TaDNAJL5**	LA+JL+AP+1C+CT	JL	TraesCS2A02G165100	TraesCS2B02G191300	TraesCS2D02G172200
**TaDNAJL6**	JL+DRD+CT	JL	TraesCS3A02G441600	TraesCS3B02G475500	TraesCS3D02G434400
**TaDNAJL7**	LA+SS+JL	JL	TraesCS4A02G118900	TraesCS4B02G185600	TraesCS4D02G186900
**TaDNAJL8**	GF+JL+DRD+1C+CT	JL		TraesCS4B02G160100	
**TaDNAJL9**	JL+DRD	JL		TraesCS4B02G618600LC	
**TaDNAJL10**	AR+JL+DRD+CT	JL	TraesCS5A02G108400	TraesCS5B02G114900	TraesCS5D02G117100
**TaDNAJL11**	JL	JL	TraesCS5A02G199900	TraesCS5B02G198200	TraesCS5D02G205500
**TaDNAJL12**	AR+JL	JL	TraesCS5A02G245400		TraesCS5D02G252200

Note: Wheat DnaJ proteins were firstly named by the homologous genes in other plants, while the others were named according to the location in chromosome. The number of wheat DnaJ proteins was sorted by homologous group. The superscript letter ‘p’ and ‘s’ represent that the HSP-encoding gene was differentially expressed in powdery mildew and stripe rust respectively.

Table S3. Wheat 70-kD heat shock proteins

Number	Gene ID in chromosome A	Gene ID in chromosome B	Gene ID in chromosome D	paralogue protein loci
**TaHsp70-1**	TraesCS1A02G120100[P]	TraesCS1B02G139500[P,S]	TraesCS1D02G121000[P,S]	
**TaHsp70-14**	TraesCS1A02G120200[S]	TraesCS1B02G139600	TraesCS1D02G121200	
**TaHsp70-6**	TraesCS1A02G133100	TraesCS1B02G151300[S]	TraesCS1D02G131800	
**TaHsp70-3**		TraesCS1B02G163400		
**TaHsp70-4**	TraesCS1A02G285000[P,S]	TraesCS1B02G294300	TraesCS1D02G284000[P,S]	
**TaHsp70-5**	TraesCS1A02G295600			TraesCS2D02G575200
**TaHsp70-7**		TraesCS2B02G125400		
**TaHsp70-8**	TraesCS2A02G506900			
**TaHsp70-9**	TraesCS2A02G507000	TraesCS2B02G535000	TraesCS2D02G625000LC	
**TaHsp70-10**	TraesCS2A02G585600		TraesCS2D02G600900	TraesCS1D02G452500
**TaHsp70-11**	TraesCS3A02G262900	TraesCS3B02G296300	TraesCS3D02G262800	
**TaHsp70-12**	TraesCS3A02G305100	TraesCS3B02G331400	TraesCS3D02G296700	
**TaHsp70-13**		TraesCS3B02G390700	TraesCS3D02G351900	
**TaHsp70-15**		TraesCS3B02G390800	TraesCS3D02G352400	
**TaHsp70-2**	TraesCS4A02G175100	TraesCS4B02G142400[P,S]	TraesCS4D02G140800[P]	
**TaHsp70-18**	TraesCS4A02G098600	TraesCS4B02G205700	TraesCS4D02G206600	
**TaHsp70-19**	TraesCS4A02G098200	TraesCS4B02G206300	TraesCS4D02G207100	
**TaHsp70-20**	TraesCS4A02G098100			
**TaHsp70-21**		TraesCS4B02G206700	TraesCS4D02G207500	
**TaHsp70-22**	TraesCS4A02G066100	TraesCS4B02G243400	TraesCS4D02G243000	
**TaHsp70-23**		TraesCS4B02G397600		TraesCSU02G116400
**TaHsp70-24**		TraesCS4B02G545200LC	TraesCS4D02G338300	TraesCS5A02G511700
**TaHsp70-25***	TraesCS5A02G078000	TraesCS5B02G087700[P]	TraesCS5D02G093900	TraesCS2B02G374700
**TaHsp70-26**	TraesCS5A02G106200	TraesCS5B02G111200	TraesCS5D02G117600	
**TaHsp70-27**		TraesCS5B02G129700		
**TaHsp70-28**	TraesCS5A02G268100	TraesCS5B02G267900	TraesCS5D02G276100	
**TaHsp70-29**	TraesCS5A02G298700	TraesCS5B02G298000		
**TaHsp70-30**	TraesCS5A02G479300	TraesCS5B02G492500	TraesCS5D02G492900[S]	
**TaHsp70-31**	TraesCS6A02G042600	TraesCS6B02G058300	TraesCS6D02G049100	
**TaHsp70-17**	TraesCS6A02G276700	TraesCS6B02G304200	TraesCS6D02G257000	

**TaHsp70-25***	TraesCS6A02G337100[P,S]	TraesCS6B02G367800[P,S]	TraesCS6D02G317700[P,S]
**TaHsp70-32**	TraesCS6A02G342200	TraesCS6B02G374500	
**TaHsp70-33**	TraesCS6A02G342400		
**TaHsp70-34**	TraesCS6A02G342500		
**TaHsp70-35**	TraesCS6A02G537000LC		
**TaHsp70-36**			TraesCS6D02G322800
**TaHsp70-37**			TraesCS6D02G339600
**TaHsp70-38**			TraesCS6D02G339700
**TaHsp70-39**			TraesCS6D02G339800
**TaHsp70-40**	TraesCS7A02G430600	TraesCS7B02G330900	TraesCS7D02G422600
**TaHsp70-16**	TraesCS7A02G457100	TraesCS7B02G359100	TraesCS7D02G445600
**TaHsp70-41**		TraesCS7B02G331400	TraesCS7D02G423100

Note: Wheat Hsp70 proteins were named by the homologous genes in other plants at first, while the others were named according to the location in chromosome. The number of wheat Hsp70 proteins was sorted by homologous group. TaHsp70-25 was repeated and marked with star symbol because the protein could be encoded by both homologous group 5 and 6 in bread wheat. The superscript letter ‘p’ and ‘s’ represent that the HSP-encoding gene was differentially expressed in powdery mildew and stripe rust respectively.

Table S4. Wheat 90-kD heat shock proteins

Number	Gene ID in chromosome A	Gene ID in chromosome B	Gene ID in chromosome D
**TaHsp90-1**	TraesCS2A02G033700	TraesCS2B02G047400	TraesCS2D02G033200
**TaHsp90-2**	TraesCS5A02G101900[P]	TraesCS5B02G106300	TraesCS5D02G113700[P]
**TaHsp90-3**	TraesCS5A02G251000	TraesCS5B02G249000	TraesCS5D02G258900
**TaHsp90-4**	TraesCS5A02G260600	TraesCS5B02G258900[P,S]	TraesCS5D02G268000
**TaHsp90-5**	TraesCS7A02G242200[P,S]	TraesCS7B02G149200[P,S]	TraesCS7D02G241100[P,S]
**TaHsp90-6**	TraesCS7A02G529900	TraesCS7B02G446900	TraesCS7D02G517800

Note: The superscript letter ‘p’ and ‘s’ represent that the HSP-encoding gene was differentially expressed in powdery mildew and stripe rust respectively.

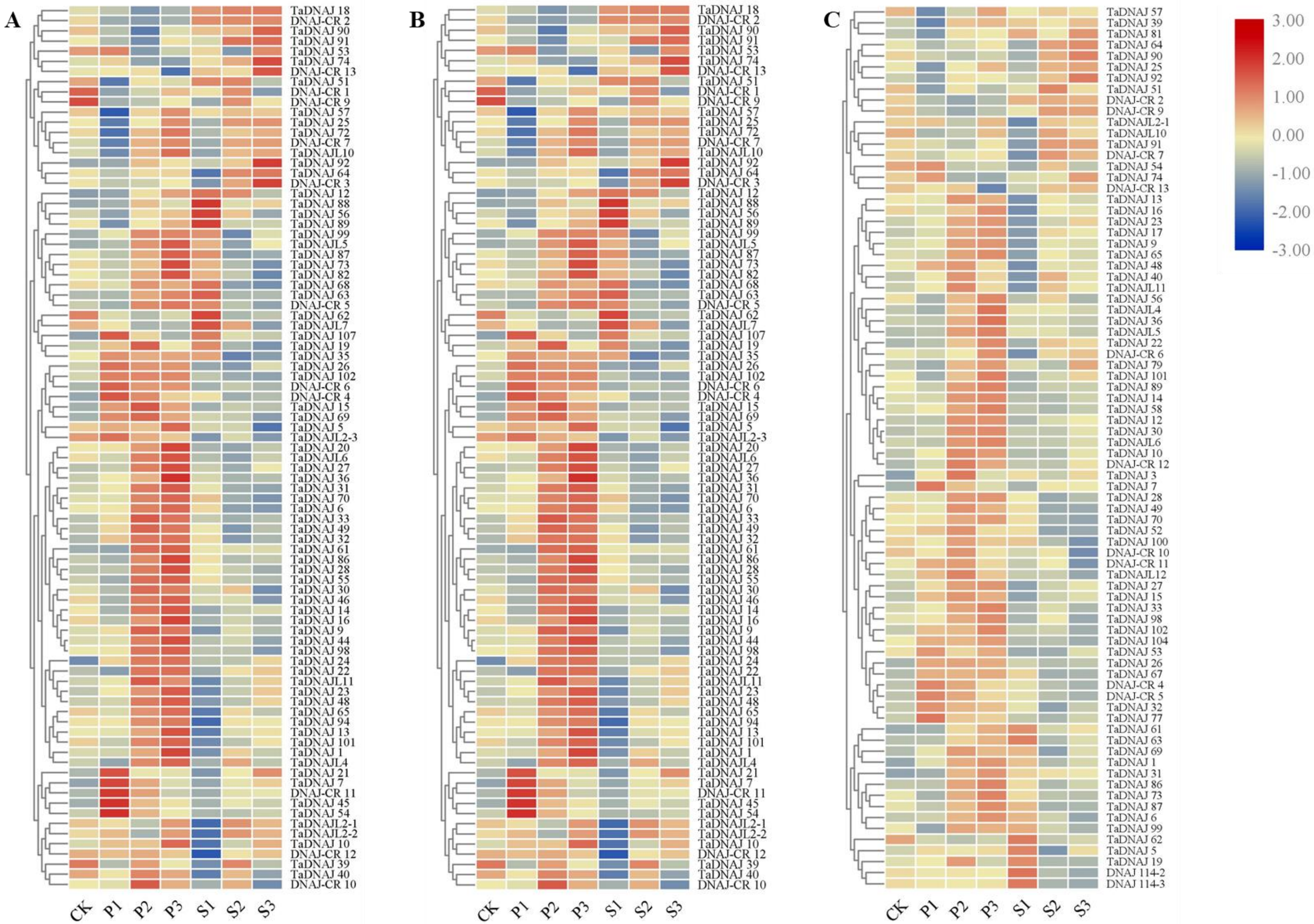

Figure S1. Expression profiles of the *TaDnaJ* genes in the three time periods of *Bgt* and *Pst* treatment. A represents *TaDnaJ* genes transcribed from A chromosome group, while B and C represent *TaDnaJs* from B and the D chromosome group, respectively. Each column (Ck, S1, S2, S3, P1, P2 and P3) represents the mean expression value of the RNA-Seq obtained from three biological replicates. Ck: resistant wheat N9134 exempt from pathogen stress as control; P1, P2 and P3: N9134 infected with powdery mildew pathogen E09 at 1, 2 and 3 dpi, respectively; S1, S2 and S3: N9134 infected with stripe rust pathogen CYR 31 at 1, 2 and 3 dpi, respectively. Color scale erected vertically at the right side of the picture represents expression values. Blue represents low level and red indicates high level of transcript abundances.

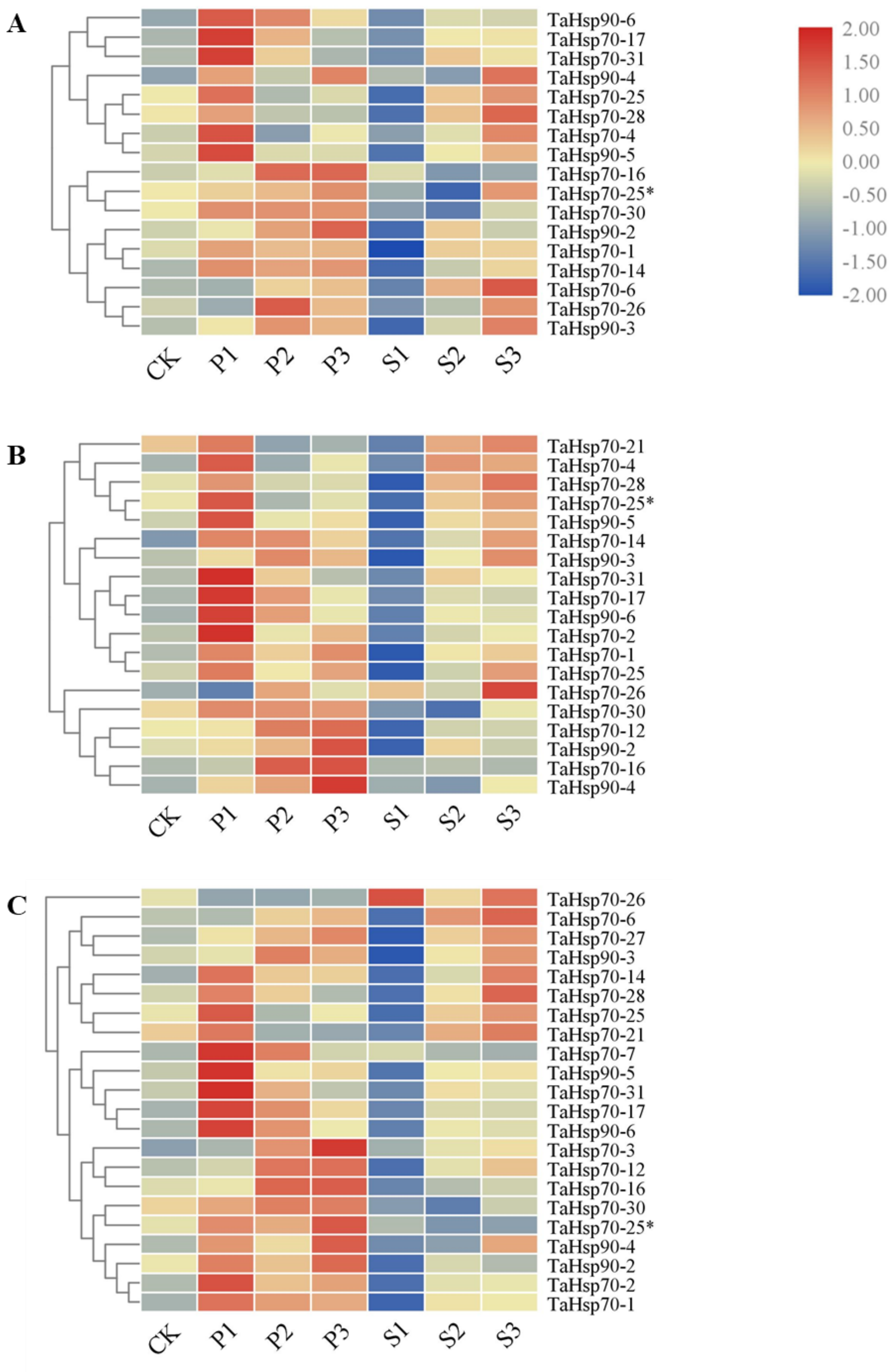

Figure S2. Expression profiles of the *TaHSP70* and *TaHSP90* genes in the three time periods of *Bgt* and *Pst* treatment. A, B and C represent *TaHSP70* and *TaHSP90* genes transcribed from A, B and D chromosome group of wheat, respectively. CK was the control without treatment. P1, P2 and P3 represent 24, 48 and 72h of *Bgt* inoculation, while S1, S2 and S3 represent 24, 48 and 72 hpi after *Pst* inoculation, respectively.

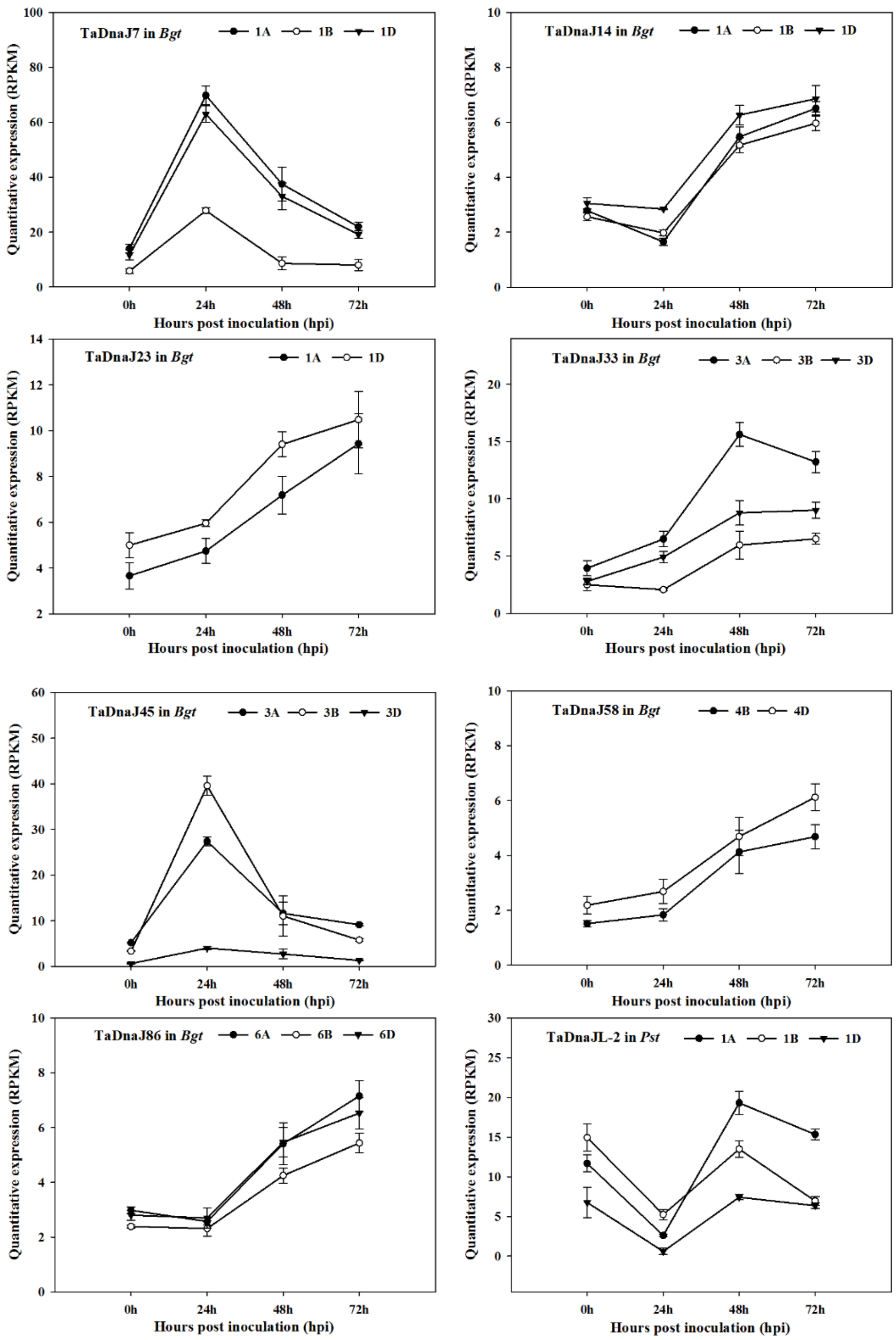

Figure S3. Expression patterns of differentially expressed *DnaJ* genes in N9134 infected by *Bgt* and *Pst*. Gene expression levels were assessed by transcript accumulation analysis. The mean expression value was calculated from three independent replicates. Line charts mean the gene expression of HSP-encoding gene in wheat infected by Pst and Bgt. The number 0, 24, 48 and 72 indicated the timepoints after infection. Bgt represents powdery mildew E09 inoculation condition; Pst represents stripe rust pathogen CYR 31 inoculation. The name of HSP-encoding genes and corresponding homologue genes were listed in the top of each panel.

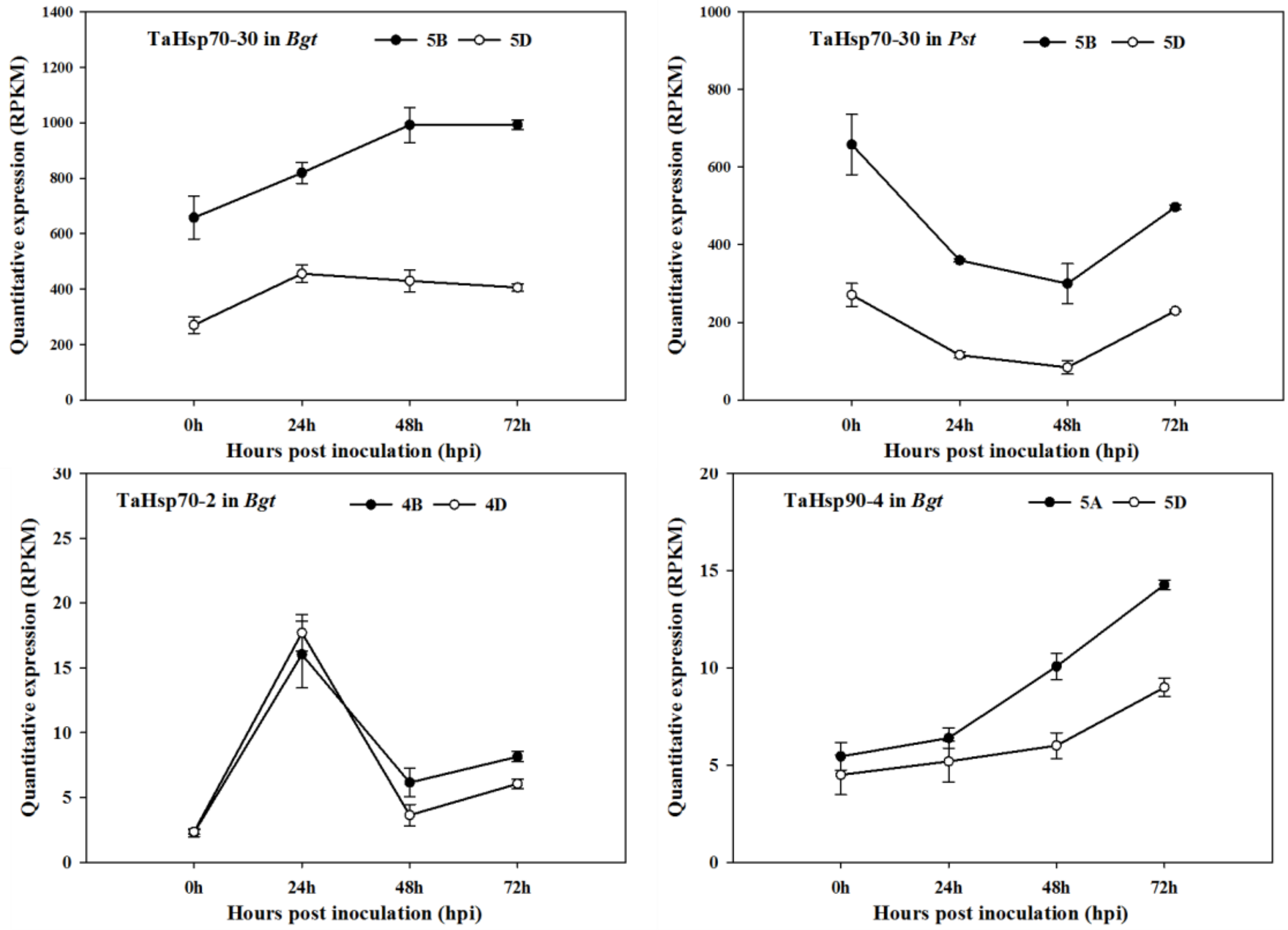

Figure S4. Expression patterns of differentially expressed *Hsp70* and *Hsp90* genes in N9134 infected by stripe rust and powdery mildew pathogen. Gene expression levels were assessed by transcript accumulation analysis. The mean expression value was calculated from three independent replicates. Line charts mean the gene expression of HSP-encoding gene in wheat infected by *Pst* and *Bgt*. The number 0, 24, 48 and 72 indicated the timepoints after infection. Bgt represents powdery mildew E09 inoculation condition; Pst represents stripe rust pathogen CYR 31 inoculation. The name of HSP-encoding genes and corresponding homologue genes were listed in the top of each panel.

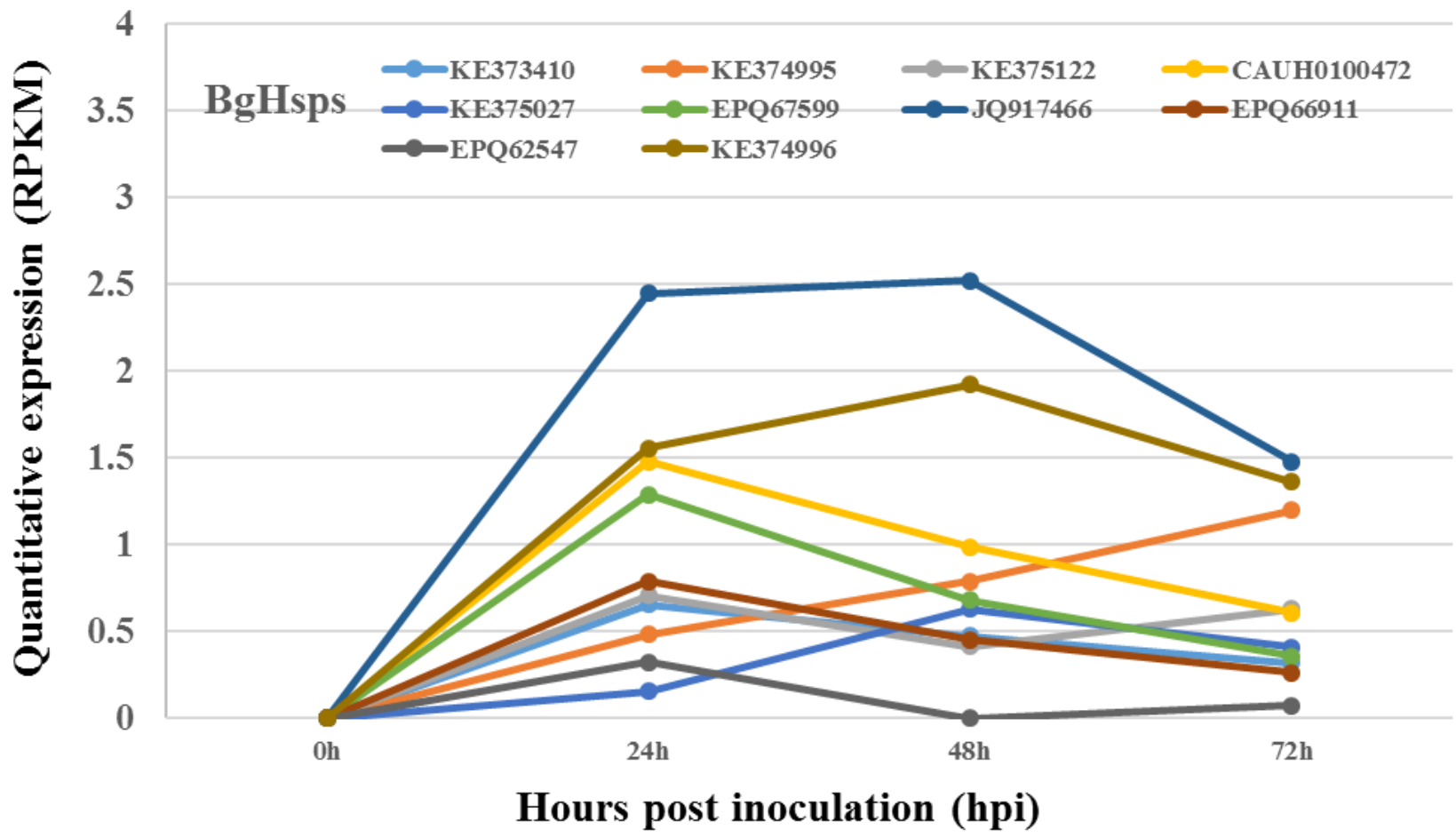

Figure S5. Relative expression of genes encoding Bgt-HSP during the wheat–*Bgt* interaction. Gene expression levels were assessed by transcript accumulation analysis. The mean expression value was calculated from three independent replicates. The X-axis indicated the timepoints at 0, 24, 48 and 72 hours after inoculation with powdery mildew Race E09. The names of homologue HSP-encoding genes of Bgt pathogen were listed in the top of panel with different color.

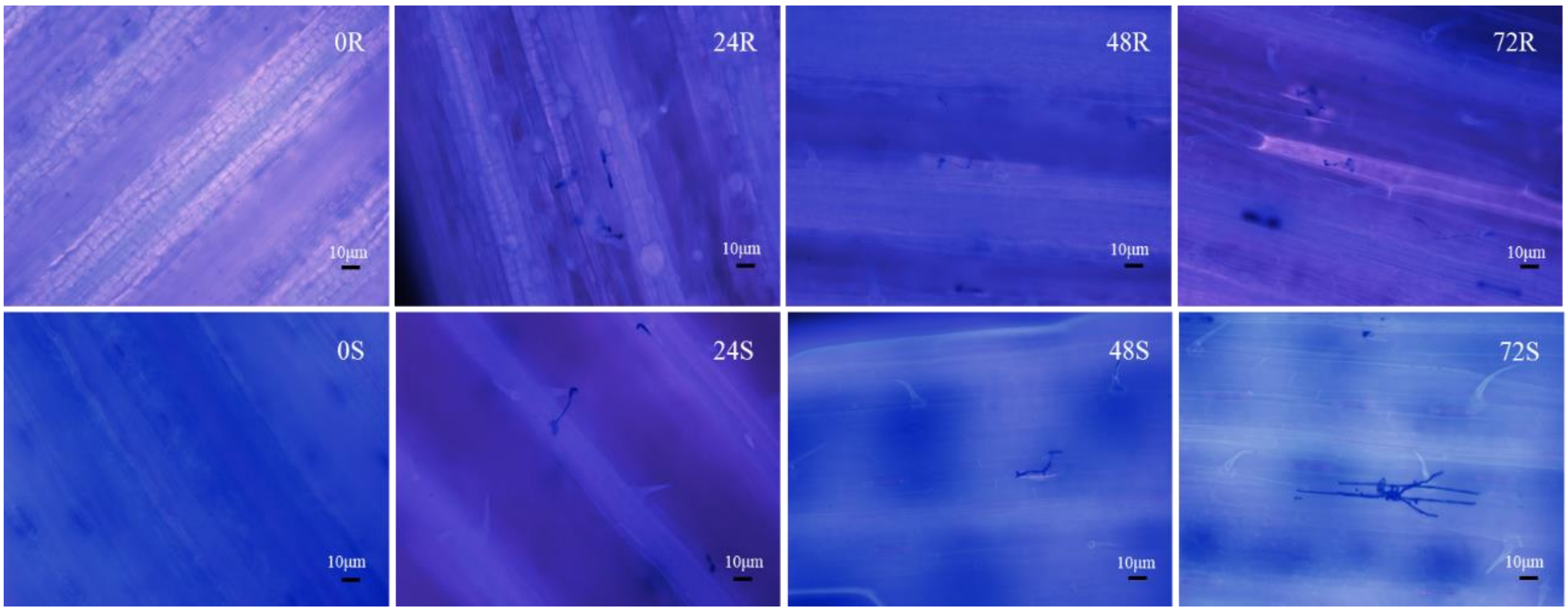

Figure S6. Cytological observations of N9134R/S-*Bgt* isolate E09 interaction. The number, 0, 24, 48 and 72 represent the time points of samplings after inoculation with *Bgt* isolate E09. R and S represent the leaf tissue of N9134R and N9134S, respectively. Pictures were photographed with an Olympus BX53 microscope.
